# The polyadenylation inhibitor cordycepin reduces pain, inflammation and joint pathology in rodent models of osteoarthritis

**DOI:** 10.1038/s41598-019-41140-1

**Published:** 2019-03-18

**Authors:** Sadaf Ashraf, Masar Radhi, Peter Gowler, James J. Burston, Raj D. Gandhi, Graeme J. Thorn, Anna M. Piccinini, David A. Walsh, Victoria Chapman, Cornelia H. de Moor

**Affiliations:** 1 School of Pharmacy, University of Nottingham, Nottingham, UK; 2 School of Life Sciences, University of Nottingham, Nottingham, UK; 3 School of Medicine, University of Nottingham, Nottingham, UK; 4 Arthritis Research UK Pain Centre, University of Nottingham, Nottingham, UK; 5 NIHR Nottingham Biomedical Research Centre, Nottingham, UK

## Abstract

Clinically, osteoarthritis (OA) pain is significantly associated with synovial inflammation. Identification of the mechanisms driving inflammation could reveal new targets to relieve this prevalent pain state. Herein, a role of polyadenylation in OA synovial samples was investigated, and the potential of the polyadenylation inhibitor cordycepin (3’ deoxyadenosine) to inhibit inflammation as well as to reduce pain and structural OA progression were studied. Joint tissues from people with OA with high or low grade inflammation and non-arthritic post-mortem controls were analysed for the polyadenylation factor CPSF4 and inflammatory markers. Effects of cordycepin on pain behavior and joint pathology were studied in models of OA (intra-articular injection of monosodium iodoacetate in rats and surgical destabilisation of the medial meniscus in mice). Human monocyte-derived macrophages and a mouse macrophage cell line were used to determine effects of cordycepin on nuclear localisation of the inflammatory transcription factor NFĸB and polyadenylation factors (WDR33 and CPSF4). CPSF4 and NFκB expression were increased in synovia from OA patients with high grade inflammation. Cordycepin reduced pain behaviour, synovial inflammation and joint pathology in both OA models. Stimulation of macrophages induced nuclear localisation of NFĸB and polyadenylation factors, effects inhibited by cordycepin. Knockdown of polyadenylation factors also prevented nuclear localisation of NFĸB. The increased expression of polyadenylation factors in OA synovia indicates a new target for analgesia treatments. This is supported by the finding that polyadenylation factors are required for inflammation in macrophages and by the fact that the polyadenylation inhibitor cordycepin attenuates pain and pathology in models of OA.

## Introduction

Osteoarthritis (OA) is a common chronic age-related joint disease, with a significant inflammatory component^1–4^, and is a leading cause of pain and disability^5^. The pathophysiology of pain in OA is complex. Treatment options are largely limited to lifestyle changes (diet and exercise) and reducing pain with non-steroidal anti-inflammatory drugs [NSAIDS] or opioids which have limited efficacy and problematic side effects. As a result, joint replacement surgery is a common outcome.

OA pathology includes synovitis, cartilage damage, osteophytes and subchondral bone changes. The most prevalent symptom of OA is pain, which is associated with inflammation^6,7^. Macrophages play a major role in driving synovitis which in turn augments the progression of OA pathogenesis^3^. The nuclear factor kappa B (NF-ĸB) family of transcription factors mediates activation of inflammatory gene expression and is upregulated in chronic inflammatory states such as OA^8^. Upon inflammatory signaling, these transcription factors translocate into the nucleus and trigger the expression of a wide range of immunomodulatory, angiogenic and proliferative factors^9^. Differentiation of osteoclasts involved in bone remodelling is also NFkB-dependent^10^.

Cordycepin (3′deoxyadenosine) is an active compound from the caterpillar fungus *Cordyceps militaris*^11^. The biochemical pathway for cordycepin is well described, once inside the cell it is converted to cordycepin triphosphate (cordyTP) which inhibits the last two steps in messenger RNA synthesis, cleavage and polyadenylation, both in nuclear extracts and tissue culture^12,13^. Incorporation of cordyTP into the poly(A) tail traps a protein complex on the incomplete mRNA. This complex includes the polyadenylation factors cleavage and polyadenylation specificity factor subunit 4 (CPSF4) and WD repeat-containing protein 33 (WDR33), as well as other proteins such as nuclear export factors^14–16^. Although it is evident that cordyTP is a polyadenylation inhibitor, other targets of cordycepin, such as adenosine receptors have been proposed^17,18^.

Previously we showed that cordycepin specifically inhibits inflammatory gene expression in human airway smooth muscle cells. The effects of cordycepin in these cells were consistent with an inhibition of polyadenylation^19^, making this process a putative target for novel anti-inflammatory drugs.

Cordycepin has effects on both cartilage and bone, reducing chondrocyte hypertrophy *in vitro* via down-regulation of runt-related transcription factor 2 (Runx2), matrix metalloproteinases (MMPS) −3 and −13 as well as a disintegrin and metalloproteinase with thrombospondin motifs **(**ADAMTS) -4 and -5^20–23^.

Both *in vitro* and *in vivo* studies support potential benefits of cordycepin treatment in preventing bone loss through inhibition of osteoclast differentiation and having osteoprotective effects during osteoporosis^24–27^. Intra-articular knee injection of cordycepin for a period of 4 to 8 weeks ameliorated cartilage damage in osteoarthritic mice^28^, however neither pain or inflammation endpoints were reported^28^.

Synovial inflammation is associated with cartilage damage and bone changes in OA, and is significantly associated with joint pain. Anti-inflammatory activity of cordycepin is evident in murine macrophages *in vitro* and attributed to the repression of NF-ĸB dependent gene expression^19,29–31^. However, it is unknown if the effects of cordycepin on inflammation in macrophages can be attributed to effects in polyadenylation or whether this is true *in vivo*.

Identification of the mechanisms driving synovial inflammation have the potential to reveal new targets to relieve OA pain. Here we investigated whether there is evidence for changes in polyadenylation factors in clinical OA synovial samples, and then the potential of the polyadenylation inhibitor cordycepin to reduce pain and structural OA progression and inflammation. Our findings identify polyadenylation as a novel target for analgesic and disease modifying drugs for OA.

## Materials and Methods

### Reagents and antibodies

All reagents were purchased from Sigma-Aldrich unless otherwise stated. The following antibodies were obtained from Abcam, MMP13 (39012), osterix (22552), VEGF (46154) and nestin (18102). DRAQ5, NFκB p65 (4764) and nestin (47607) antibodies were purchased from Cell Signalling Technology Inc. WDR33 (374466) and ADAMTS5 (83186) antibodies were purchased from Santa Cruz Biotech. CPSF4 antibody was obtained from Protein Tech. PCNA (M0879) and CD68 (M0814) antibodies were obtained from Dako. Alkaline phosphatase and peroxidase kits as well as secondary antibodies were obtained from Vector Labs. MCSF was obtained from R&D Systems. RANKL was obtained from Peprotech.

### Rodent models of OA

Studies were in accordance with UK Home Office Animals (Scientific Procedures) Act (1986) and the International Association for the Study of Pain guidelines and were approved by ethical review board at the University of Nottingham. Data are presented in line with the ARRIVE guidelines. All animal studies were conducted in a manner that minimised animal distress, and euthanization of the animal occurred via an appropriate S1 technique (as listed by the UK Home Office). Animals were anesthetised with isoflurane (2.5–3%) in 100% oxygen (1 L per min) prior to surgeries and intra-articular injections. Tissues including synovia and joints were collected for molecular biology, histological and immunohistochemistry studies.

Male Sprague Dawley rats (n = 10/group) weighing 180–200 g were given intra-articular injection of monosodium iodoacetate (MIA) (1 mg/50 μl) in saline at day 0 into their left knee^32^. Control rats received intra-articular injection of 50 µl of saline. For the therapeutic MIA study, at Day 14, cordycepin (4 mg/kg, 8 mg/kg or 16 mg/kg) or vehicle (1 ml distilled water) were mixed with 1 g of wet mash and administered every other day until day 28. For the pre-emptive MIA study, cordycepin (8 mg/kg) was given at day 0 (prior to intra-articular injection) for a period of 2 weeks, until day 14. Rats were food restricted for 2 hrs prior to being given cordycepin. Pain behaviour was measured twice weekly following model induction.

Eight to nine weeks old male C57BL/6 mice (at least n = 15/group) underwent surgery on their left knee joint at week 0 to displace the medial meniscus as described previously^33^. A small longitudinal incision was made over the joint and using blunt dissection the underlying medial meniscotibial ligament (MMTL), which anchors the medial meniscus to the tibial plateau was transected, destabilising the medial meniscus (DMM). The wound was sutured and mice observed until they regained consciousness. Control group included those having sham surgery, in which the ligament was visualised but not transected. From week 14 to 16 mice were orally gavaged every other day with 200 µl cordycepin (8 mg/kg) or vehicle (23% propylene glycol [PPG] in distilled water)^34^. Pain behaviour was measured once weekly following model induction and then twice weekly following cordycepin treatment, until week 16.

Pain behaviour was quantified as a change in hindlimb weight-distribution and hindpaw mechanical withdrawal thresholds, as previously described^32^.

### Human osteoarthritic and post-mortem joint tissues

The joint tissue repository of the Arthritis Research UK Pain Centre, which contains samples from >1,700 subjects, was screened to select tissues obtained at the time of total knee replacement (TKR) for OA and tissues obtained post-mortem from age-matched subjects who had not sought medical attention for knee pain during the last year of life (non-OA control group). The tissue samples (n = 10 per group) were split into three distinct groups, OA group having high grade inflammation (median age [IQR]; 58 [57–73], 78% were male), OA group having low grade inflammation (median age [IQR]; 61 [58–70], 67% were male) and non-OA control group (median age [IQR]; 60 [50–70], 67% were male). Synovial inflammation was graded on a scale of 0–3 (where 0 = normal and 3 = severe [high grade] inflammation) by assessing the degree of synovial lining hyperplasia, inflammatory cell infiltrate, and cellularity^35^. Patients undergoing TKR fulfilled the American College of Rheumatology classification criteria for OA at the time of surgery^36^. Subjects from whom samples were obtained postmortem were recently deceased, had no history of rheumatoid arthritis or pseudogout, and had not previously sought help for knee pain during the last year of life, as determined by interviews with the relatives and review of case notes. Exclusion criteria for non-OA controls consisted of a history of OA, Heberden’s nodes identified on clinical examination, macroscopic chondropathy lesions of grade 3 or 4 in the medial tibiofemoral compartment, or osteophytes on direct visualization of the dissected knee. Informed consent was obtained from the TKR patients and from the next of kin of the postmortem subjects. All study protocols were performed in accordance with the relevant guidelines and regulations indicated by the UK National Research Ethics Service (Nottingham Research Ethics Committee 1 [05/Q2403/24] and Derby Research Ethics Committee 1 [11/H0405/2]).

### Tissue processing

Rat synovia with patellae were dissected, embedded in OCT and snap frozen in isopentane. Tibiofemoral joints were fixed for 48 hrs in 4% paraformaldehyde (PFA), then decalcified in 10% ethylenediaminetetraacetic acid (EDTA) in 10 mM Tris buffer (pH 6.95) for 4 weeks on a shaker at room temperature (RT). Coronal sections of trimmed joint tissues were mounted in paraffin wax. Mice synovia with patellae and tibiofemoral joints were either frozen on dry ice or the whole knee joints were fixed in 4% paraformaldehyde for 24 hrs before being decalcified in EDTA for 6 days on a shaker at RT. Sagittal sections of trimmed joint tissues were mounted in paraffin wax. For human tissue samples, midcoronal sections of the middle one-third of the medial tibial plateau were fixed in neutral-buffered formalin and then decalcified in 10% EDTA in 10 m*M* Tris buffer (pH 6.95; at 4 °C) prior to embedding in wax. Surgeons and technicians were instructed to collect synovium from the medial joint line. Synovial tissues were fixed in formalin and embedded in wax without decalcification.

### Joint histology

All sections for histology were cut at 5 μm and visualised using a 20 × objective lens unless otherwise indicated. All histomorphometry analysis was done on haematoxylin and eosin or Safranin-O/fast green-stained sections by at least two observers blinded to the treatment groups.

In the rat MIA model, cartilage damage, matrix proteoglycan and osteophytes were assessed as previously described^37^. The integrity of the osteochondral junction (OCJ) was measured as the number of channels (and those that were nestin positive) crossing the OCJ into the cartilage of the whole section of medial tibial plateau^37^. Synovial inflammation was graded as previously described^38^ on a scale from 0 (lining cell layer 1–2 cells thick) to 3 (lining cell layer >9 cells thick and/or severe increase in cellularity).

In the mice DMM model, joint pathology was assessed based on previously published scoring criteria^39,40^. Briefly, cartilage surface integrity was scored from 0 (normal) to 6 (vertical clefts/erosions to the calcified cartilage extending to >75% of the articular surface). Cartilage proteoglycan loss was scored from 0 (normal staining of non-calcified cartilage) to 5 (complete loss of safranin-o/fast green staining in the non-calcified cartilage extending to ≥75% of the articular surface). Chondrocyte hypertrophy score ranged from 0 (no chondrocyte hypertrophy) to 1 (enlarged chondrocyte lacunae with lack of safranin-o/fast green stain). Osteophyte size was scored from 0 (no osteophyte) to 3 (large osteophyte, greater than 3 x the thickness of the adjacent cartilage. Osteophyte maturity scores ranged from 0 (no osteophyte) to 3 (predominantly bone). Subchondral bone thickening score ranged from 0 (normal trabecular bone with greater than 50% marrow space) to 3 (solid bone spanning greater than two thirds of the width of the epiphysis). Synovial inflammation was graded on a scale of 0 (no inflammation: lining cell layer 1–2 cells thick) to 3 (severe inflammation: greater than 6 cells thick lining). In the human synovial sections inflammation was graded on a scale of 0–3 (where 0 = normal and 3 = severe inflammation) by assessing the degree of synovial lining hyperplasia, inflammatory cell infiltrate, and cellularity^35^.

### Immunohistochemistry and immunofluorescence

Synovial inflammation was measured as CD68 (clone ED1) positive macrophages as previously described^41^. Proliferating cell nuclear antigen (PCNA) positive cells and PCNA-immunoreactive CD31-positive cells were used to identify proliferating cells and proliferating endothelial cells respectively, as measures of the extent of synovial proliferation and angiogenesis^41^. To detect ADAMTS5, MMP13, nestin, VEGF, PCNA, CD68, NF-ĸB and CPSF4 immunoreactivity in paraffin embedded tissue sections, the sections were first deparaffinised and rehydrated in graded ethanol and water, followed by antigen unmasking (10 mM sodium citrate buffer, pH 6) at 80–85 °C for 20 mins. Sections were cooled for 10 mins at RT followed by permeabilisation (0.1% Triton X-100) and blocking (5% serum) steps. Primary antibodies were incubated overnight at 4 °C and secondary antibodies for 45 mins at RT. Vectastain ABC-AP alkaline peroxidase with Fast Diaminobenzidene (DAB) was used to visualise ADAMTS-5, MMP13, nestin, VEGF, CPSF4 and PCNA staining. Preparations were mounted in DePeX. To detect NF-ĸB, WDR33, CPSF4 and CD68 immunofluorescence in tissue sections and cell cultures, Alexa Fluor 488 and 568 secondary antibodies were used. Cell cultures were fixed for 15 mins in 4% PFA before proceeding to the immunofluorescence protocol as described above. DRAQ5 was used as nuclear stain and sections were mounted in aqueous mounting media.

### Osteoclast number

Tissue sections were dewaxed and recalcified before tartrate-resistant acid phosphatase (TRAP) staining. The number of TRAP-positive multinucleated osteoclasts were quantified within the subchondral bone area comprising the area between the cartilage/bone junction and the growth plate as described previously^42^.

### *In-vitro* model of human macrophage and osteoclast differentiation

This study was approved by the Nottingham University Medical School Research Ethics Committee. Monocytes were isolated from peripheral blood of healthy human donors and either differentiated into macrophages or osteoclasts as previously described^43^. For osteoclast differentiation, monocytes were isolated from buffy coats by gradient centrifugation and seeded onto glass coverslips within a 24-well culture plates, and cultured in growth media supplemented with human macrophage colony stimulating factor (MCSF) and human receptor activator of NF-ĸB ligand (RANKL), unless otherwise stated. Cells were incubated at 37 °C, 7% CO^2^ for 2 hrs, and the medium replaced. Growth media containing cordycepin (20 µM) was then added to the cells. After 14 days, cells were washed and fixed with 4% PFA. Differentiated osteoclasts were identified by TRAP staining. For quantification of TRAP positive cells five random fields of view were counted per coverslip using four coverslips per condition. Cells that stained positive for TRAP and had three or more nuclei were counted. For macrophage differentiation, the monocytes were grown in RPMI 1640 supplemented with 5% foetal bovine serum (FBS) in the presence of MCSF for 5 days. Adherent cells were washed, replated onto coverslips in a 24-well plate and cultured for a further 24 h in 3% FBS before stimulation with LPS with and without cordycepin. Cells were fixed in 4% PFA before proceeding to the immunofluorescence protocol. RAW264.7 cells were maintained in Dulbecco’s Modified Eagle Medium (DMEM) with 10% FBS in a humidified atmosphere of 5% CO_2_ and 95% air at 37 °C. Twenty four hours before experimentation, the cells were washed with PBS and supplemented with 0.5% serum. The cells were then treated with cordycepin (20 µM) either 1 hr before or 10 mins after LPS stimulation (1 µg/ml). After which the cells were either processed for protein/RNA extraction or immunofluorescence protocol. RNA isolation for tissue culture cells was done using the Promega Reliaprep system.

### Western blot analysis

RAW264.7 cells were lysed with radioimmunoprecipitation assay (RIPA) buffer (0.5% Igepal, 0.5% deoxycholate, 0.05% sodium dodecyl sulfate, 1 mM β-glycerophosphate, 1 mM Na_3_VO_4_, 1 mM phenylmethylsulfonyl fluoride) containing protease/phosphatase inhibitors to extract total cell protein content. Protein concentration was determined by Bradford Assay. Approximately 30 µg of protein was subjected to SDS-PAGE and transferred to nitrocellulose membrane. To block non-specific binding of proteins, membranes were treated in TBST with 5% skimmed milk for 1 hr at RT, and were incubated overnight with primary antibodies against IĸB and vinculin at 4 °C, followed by secondary antibody incubation for 1 hr at RT. Immunoreactivity was detected by chemiluminescence. Western blot images were not reassembled after cuts between the vertical lanes. The blots were cut horizontally so to make it easier to probe for various antibodies simultaneously on the same blot. Each continuous image represents a single exposure.

### RNA isolation from tissues

At the end of the pain behavioural studies conducted in the DMM-model of OA, fresh-frozen synovial tissue and knee joints were collected and stored at −80 °C. Tissues were homogenized using the bullet blender. Total RNA was extracted from the tissues and RAW264.7 cells using TRIzol.

### Quantitative real-time polymerase chain reaction (qRT-PCR)

500 ng of RNA was reversed transcribed to cDNA and diluted 5 fold with sterile distilled water before being subjected to qPCR using the GoTaq qPCR Master Mix containing the relevant forward and reverse primer sets (Supplementary Table 1). Primers for CD68, IL1β (spliced and unspliced), nestin, VEGF, PCNA, MYC, osterix, RUNX1, RUNX2 and TNF (spliced and unspliced) were designed with Primer Express 3.0 software. All qRT-PCR experiments were performed in triplicates, data were normalised to relative expression using the Qiagen Rotor-Gene Q software. All values were normalised to Ribosomal Protein L28 (RPL28) using the 2^−∆∆ct^ method.

### siRNA Transfection Protocol

RAW264.7 cells were transfected for 24 hrs with lipofectamine diluted in opti-MEM containing 5 nM siRNA for either WDR33 (Dharmacon SMARTpool ON-TARGETplus L-051645-01-0005) or CPSF4 (Dharmacon SMARTpool ON-TARGETplus L-052851-01-0005). Media was changed the next day and cells transfected again and incubated for a period of 24 hrs before being processed for either immunofluorescence or RNA extraction.

### Microarray analysis

High-throughput analysis was conducted on RNA extracted from RAW 264.7 cells treated with either 20 μM cordycepin or vehicle control for 1 hr before being stimulated with LPS (1 μg/ml) or vehicle control for a further 1 hr, to reveal the genome wide changes brought about by cordycepin. Biological replicates (n = 4, 16 RNA samples in total) were then analysed on a mouse GE 8 × 60 K microarray (Agilent, cat no G4852A). This was followed by cluster analysis on the lists of RNAs whose levels were changed by cordycepin. This gene ontology analysis was done using the Database for Annotation, Visualization and Integrated Discovery (DAVID) v6.8 software on the list of genes most strongly downregulated in the LPS with cordycepin treatment group compared to LPS alone group.

### Image analysis and quantification

Synovial macrophage fractional area was the percentage of synovial section area positive for CD68, and synovial angiogenesis was measured as endothelial cell proliferation index, defined as the percentage of endothelial nuclei positive for PCNA, derived using four fields per section and one section per case as described previously^2,44^. Cartilage cellularity was quantified by counting the chondrocytes in three microscopic fields (355 μm x 265 μm) per section, at central, medial and lateral side of the medial tibial plateau (MTP) taken under 40 × magnification. Total number of chondrocytes and number of positively stained chondrocytes were counted in each section. Results were expressed as the percentage positive cells. At least 3 images per section and 3 sections per block were analysed^45^. Nestin and VEGF expression in the subchondral bone was analysed using imageJ software as the area (µm^2^) covered by nestin/VEGF immunoreactivity. PCNA expression in human synovial tissues was analysed on deconvoluted DAB and haematoxylin images in ImageJ as percentage of PCNA positive cells. NF-ĸB immunofluorescence was analysed on ImageJ as nuclear versus cytoplasmic intensity expression or intensity per µm^2^. All histology and immunohistochemistry image analyses were performed using a Zeiss Axioskop 50 microscope and a KS300 image analysis system. Immunofluorescence images were captured using a Leica confocal microscope.

### Statistical analysis

Data were analyzed with GraphPad Prism version 6 and are presented as either the mean ± SEM or mean ± SD. For all comparisons, p < 0.05 was taken to indicate statistical significance. Parametric data were analysed using analysis of variance (ANOVA) with post hoc Dunnett’s test. Univariate comparisons were made against controls using the Student t test. Non-parametric data were analysed using the Kruskal–Wallis test followed by the Mann–Whitney test with Bonferroni correction.

## Results

### The polyadenylation marker CPSF4 is elevated in inflamed synovial tissues from people with OA

**Figure 1:**
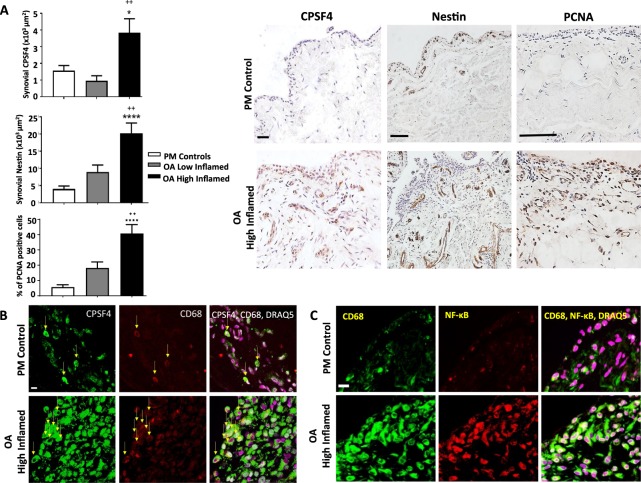
Expression of CD68 positive macrophages, cell proliferation, angiogenesis, NFκB and the polyadenylation marker CPSF4 in synovia from OA patients with varying degree of inflammation. Synovia from OA patients with high grade inflammation (n = 10), low grade inflammation (n = 10) and post-mortem (PM) control tissues (n = 10) were used to detect the expression of the polyadenylation marker CPSF4, angiogenesis marker nestin, extent of cell proliferation (proliferating cell nuclear antigen [PCNA], CD68 positive macrophages and NFκB. Synovia from OA patients with high grade inflammation expressed higher levels of CPSF4 (**A**,**B**) and NFκB (**C**) compared to PM non-arthritic control tissues, and the expression was localised to CD68 positive macrophages. CPSF4 expression was seen in the nucleus and cytoplasm of CD68 positive macrophages (**B**) as indicated by yellow arrows. Greater density of nestin positive blood vessels (**A**) and increased cell proliferation (**A**) was also seen in the synovia from OA patients with high grade inflammation compared to PM control tissues. Synovia from OA patients with low grade inflammation were similar to PM control tissues. DRAQ5 was used to detect cell nuclei. Scale bar = 20 µm. Data are mean ± SEM of tissues from n = 10 patients per group. *p < 0.05 versus post mortem controls. ^++^p < 0.01 versus low inflamed OA group.

Increased synovial cellularity and angiogenesis were observed in synovia from people with OA who had high grade synovial inflammation compared with age and sex-matched post mortem non-arthritic control tissues (Fig. 1A). Synovia from people with OA with low grade inflammation were similar to post mortem control tissues. Increased NFκB and CPSF4 expression observed in synovia from people with OA who had high grade inflammation was localised to CD68 positive macrophages (Fig. 1B,C). These data demonstrate that there are significant changes in the polyadenylation machinery in human OA synovitis.

### Cordycepin reduces pain and synovial inflammation in two rodent OA models

To assess the potential of cordycepin as a treatment for OA we used two different rodent OA models.

**Figure 2:**
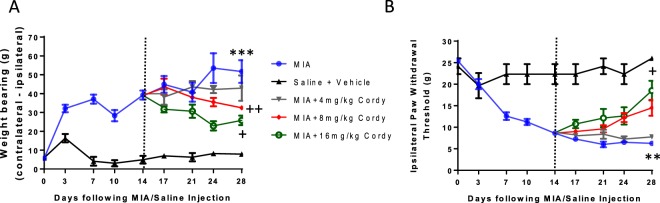
Cordycepin reduces established pain behavior in a dose-dependent manner in the MIA model of OA. Male Sprague Dawley rats (n = 8/group) were given intra-articular injection of MIA (1 mg/50 μl) at day 0. At Day 14 (dotted line), cordycepin or vehicle mixed with 1 g of wet mash was administered every other day until day 28. Rats were food restricted for 2 hrs prior to being given cordycepin. At the time of being given cordycepin, rats were moved to individual cages (housed 1/cage) and given wet mash containing cordycepin. Rats were habituated for pain behaviour testing (**A**: incapacitance [weight-bearing] and B: Von-Frey [paw-withdrawal threshold]) prior to model induction. Pain behaviour was measured twice weekly following model induction until day 28. Pain behavior increased in the arthritic animals (blue line) following MIA injection (day 0) compared to controls (black line) and the increase was maintained until day 28. Dose dependent reduction in pain behavior is evident following cordycepin administration at day 14. With higher doses of cordycepin being more effective at reducing pain behavior. *versus saline + vehicle treated groups. ^+^versus MIA group.

Oral doses of 8 mg/kg and 16 mg/kg cordycepin were similarly effective at reducing pain behaviour in rats with OA induced by intra-articular injection of MIA and 8 mg/kg was used for all further animal studies (Fig. 2).

**Figure 3:**
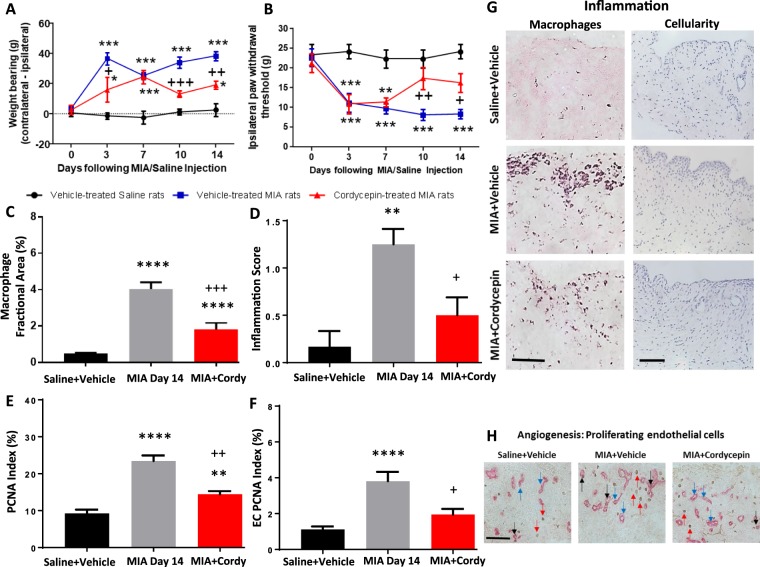
Pre-emptive cordycepin treatment reduces pain and synovial inflammation in the MIA model of OA. OA was induced on day 0 by injecting MIA (1 mg/50 µl) in the left knee joints of male Sprague Dawley rats (**A**,**B**). Cordycepin (Cordy; 8 mg/kg, orally, every other day) or vehicle (Veh) was administered for a period of 2 weeks, starting at day 0 until day 14. Saline (50 µl) injected rats were used as controls. Cordycepin treatment reduced MIA-induced changes in pain behaviour measured as weightbearing asymmetry (**A**) and mechanical allodynia (**B**). Cordycepin treatment reduced MIA-induced increase in synovial macrophages (**C**,**G**), cellularity/lining thickness (**D**,**G**), cell proliferation (**E**,**H**) and angiogenesis (F and H). Immunostaining of CD68 positive macrophages and haematoxylin and eosin stained sections showed cellular infiltration (**G**). Immunostained sections for proliferating endothelial cells (ECs) (proliferating cell nuclear antigen [PCNA] positive CD31 cells); black arrows, non-proliferating ECs; blue arrows and PCNA positive cells; red arrows (H). Data are presented graphically as mean ± SEM from n = 10 rats/group. *p < 0.05, **p < 0.01, ***p < 0.001, ****p < 0.0001 versus vehicle-treated saline-injected controls. ^+^p < 0.05, ^++^p < 0.01, ^+++^p < 0.001, ^+++^p < 0.0001 versus vehicle-treated MIA-injected day 14 OA rats.

Preventative oral cordycepin treatment given 2 hrs prior to MIA injection and thereafter every other day for 2 weeks (day 0 to 14) reduced MIA-induced pain behaviour measured as hind limb weight bearing asymmetry and ipsilateral paw withdrawal threshold (Fig. 3A,B). MIA-induced synovitis (Fig. 3C,D,G), synovial cell proliferation (Fig. 3E,H) and synovial angiogenesis (Fig. 3F,H) were also reduced following cordycepin treatment. Initial reduction in pain behaviour following preventative treatment with cordycepin in the MIA model was seen at day 3 and this reduction was maintained through day 14 (Fig. 3A,B).

**Figure 4:**
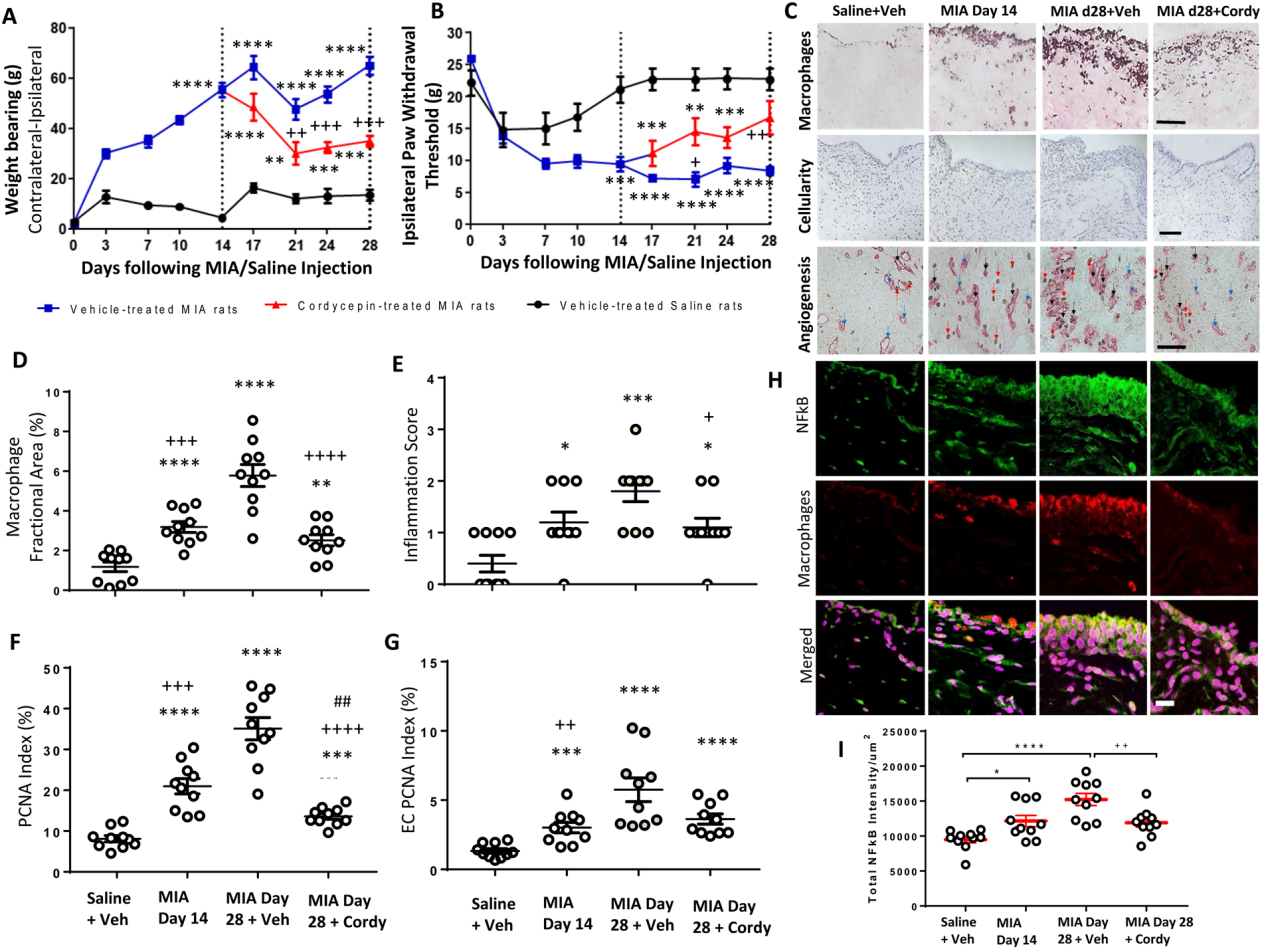
Therapeutically administered cordycepin reduces MIA-induced pain and synovial inflammation. OA was induced on day 0 by injecting MIA (1 mg/50 µl) in the left knee joints. Cordycepin (Cordy; 8 mg/kg, orally, every other day) or vehicle (Veh) was administered for a period of 2 weeks starting at day 14; once MIA-induced changes were established, to day 28. Saline (50 µl) injected rats were used as controls. Cordycepin treatment reduced MIA-induced changes in pain behaviour measured as weightbearing asymmetry (**A**) and mechanical allodynia (**B**). Synovial sections were immunostaining for CD68 positive macrophages, PCNA positive CD31 blood vessels and proliferating endothelial cells (ECs) (proliferating cell nuclear antigen [PCNA] positive CD31 cells); black arrows, non-proliferating ECs; blue arrows and PCNA positive cells; red arrows (**C**). Haematoxylin and eosin stained sections showed cellular infiltration (C). MIA-induced synovial inflammation measured as macrophage fractional area (**D**) and synovial lining thickness score (**E**) as well as synovial cell proliferation (**F**) were also reduced in the cordycepin treated groups. Cordycepin treatment did not significantly reduce MIA-induced synovial angiogenesis (**G**). Immunofluorescence staining for synovial NFκB (p65) intensity (**H**,**I**) showed that synovial macrophages expressed NFκB and the expression of NFκB increased with MIA-induced disease progression (**H**). Cordycepin treatment reduced the synovial NFκB (p65) intensity (I). Scale bars are 100 µm. Data are mean ± SEM of n = 10 rats per group. *p < 0.05, **p < 0.01, ***p < 0.001, ****p < 0.0001 versus vehicle-treated saline-injected controls. + p < 0.05, ^++^p < 0.01 versus vehicle-treated MIA-injected OA rats at day 28. ^##^p < 0.01 versus MIA-injected OA rats at day 14.

When cordycepin was given therapeutically to rats with MIA-induced OA for a period of 2 weeks (day 14 to 28; every other day) it first reduced the pain behaviour as measured by the hind limb weight bearing asymmetry and ipsilateral paw withdrawal threshold (Fig. 4A,B) at day 21 (7 days after cordycepin treatment). This decrease in pain behaviour was maintained through day 28. The reduction in MIA-induced pain behaviour was accompanied by a reduction in MIA-induced increases in synovial macrophages (Fig. 4C–E), angiogenesis (Fig. 4C,G), cellularity (Fig. 4C,F) and NFκB expression by synovial macrophages (Fig. 4H,I). Although CPSF4 expression was somewhat increased in the synovia from the MIA model of OA, neither this nor inhibitory effects of cordycepin on CPSF4 expression reached statistical significance (Supplementary Fig. 1).

**Figure 5:**
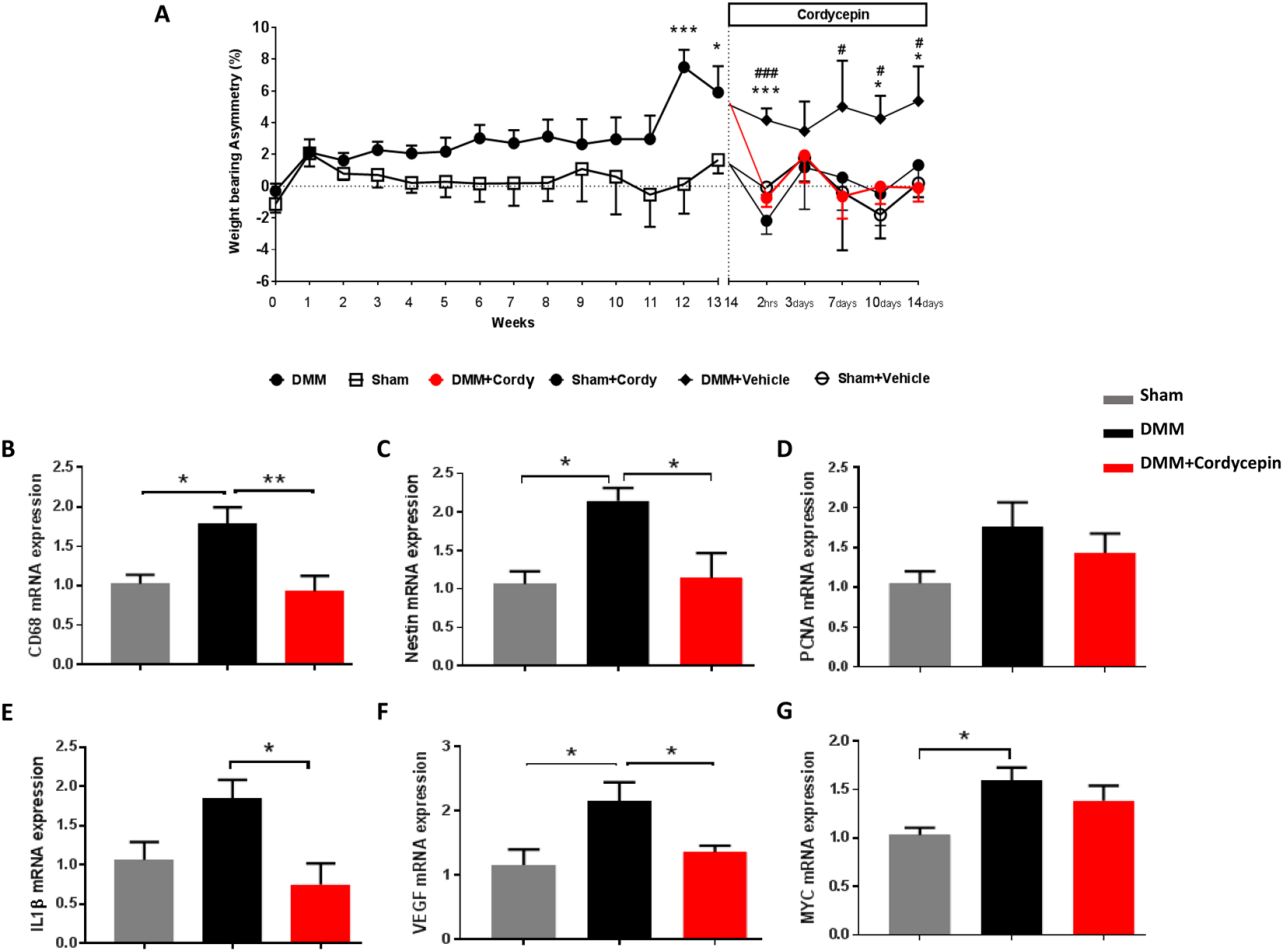
Cordycepin treatment reduces established knee joint pain and synovial inflammation in the DMM model of OA. OA was induced on day 0 by surgically displacing the medial meniscus (DMM). Sham-operated mice were used as controls in which the ligament was visualised but not transected. Cordycepin or vehicle was given orally for a period of 2 weeks, starting at week 14 when knee joint pain measured as weightbearing asymmetry (**A**) was first evident in the DMM operated mice. Sham-operated mice did not exhibit joint pain. Cordycepin (cordy) treatment reduced the established pain behavior observed following DMM surgery (**A**). Fresh frozen synovial tissues were dissected at week 16 and synovial tissue mRNA expression analysed for inflammatory (**B**; CD68 and E; IL1β) and angiogenic (**C**; nestin and **F**; VEGF) markers with no alteration in cell proliferation (D; PCNA and G; MYC) markers. Data are mean ± SEM of n = 15 mice per group. *p < 0.05, **p < 0.01, ***p < 0.001, ****p < 0.0001 versus sham controls. ^#^p < 0.05, ^##^p < 0.01, ^###^p < 0.001 versus DMM + Cordy group.

Induction of the DMM model in mice exhibited a significant increase in weight bearing asymmetry by week 12 and this was maintained through week 16 (Fig. 5A). DMM surgery did not significantly alter paw withdrawal threshold (Supplementary Fig. 2). Cordycepin treatment (every other day from week 14 to 16) reduced established pain behaviour measured as hind limb weight bearing asymmetry in the DMM model (Fig. 5A). Initial reduction was seen 2 hrs following oral cordycepin and maintained through week 16 (Fig. 5A). The DMM model was also associated with synovial inflammation (Fig. 5B,C and Table 1), and with increases in synovial mRNA expression levels of inflammatory (Fig. 5B,E), angiogenesis (Fig. 5C,F) and cell proliferation (Fig. 5D,G) markers. A 2 week oral treatment with cordycepin reduced synovial mRNA expression of inflammatory (CD68 and IL1β) and angiogenic (nestin and VEGF) markers in the DMM model, indicating effects of cordycepin on synovial inflammation at the cellular level, although significance was not achieved for cell proliferation (PCNA and MYC) markers (Fig. 5D,G), nor on histological synovial inflammation score (Table 1).

**Table 1:** Cordycepin treatment reduces cartilage and bone damage in the mice destabilising of medial meniscus (DMM) model of OA. Values are the median (IQR). ^+^p < 0.05, ^++^p < 0.01 versus arthritic (DMM) mice.

Structural changes in the knee joint (Histology Score)	Treatment Groups		
	Non-arthritic	Arthritic (DMM)	DMM + Cordycepin
Cartilage Damage (range 0–6)	0 (0–0.25)^++^	3 (2.5–4.5)	0.25 (0–1.25)^+^
Chondrocyte Hypertrophy (range 0–1)	0 (0–1)^++^	1 (1–1)	0 (0–0.25)^+^
Proteoglycan Loss (range 0–5)	0 (0–0.3)^++^	1.5 (0.5–1.5)	0 (0–0.25)^++^
Osteophyte Size (range 0–3)	0 (0–0.1)^+^	0.5 (0.5–1)	0 (0–0.1)^+^
Osteophyte Maturity (range 0–3)	0 (0–0)^++^	1 (0.5–1.5)	0 (0–0.1)^+^
Subchondral Bone Thickness (range 0–3)	0 (0–0.6)	1 (0.5–3)	0 (0–0.4)^+^
Synovial Inflammation (range 0–3)	0.5 (0–1.1)^+^	2 (1–3)	2 (0.5–2)

### Effects of cordycepin on cartilage damage and subchondral bone changes in two rodent OA models

**Figure 6:**
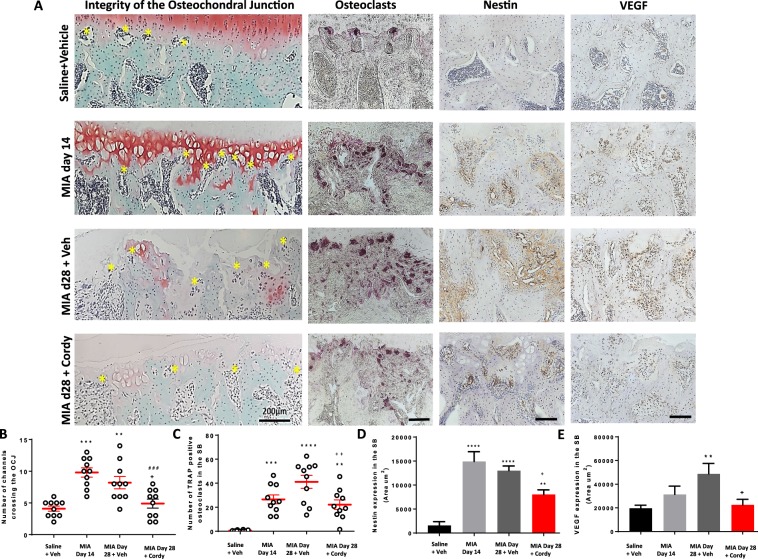
Cordycepin treatment reduces monosodium iodoacetate (MIA)-induced subchondral bone changes. OA was induced by injecting MIA (1 mg/50 µl) in the left knee joints on day 0. At day 14, cordycepin (Cordy; 8 mg/kg, orally, every other day) or vehicle (Veh) was administered for a period of 2 weeks until day 28. Saline (50 µl) injected rats were used as controls. Cordycepin treatment reduced MIA-induced increase in number of channels crossing the osteochondral junction (OCJ) (**A**,**B**), tartrate-resistant acid phosphatase (TRAP) positive osteoclasts (**A**,**C**) as well as nestin (**A**,**D**) and VEGF (**A**,**E**) expression in the subchondral bone. Vehicle-treated saline-injected control rats showed fewer number of channels crossing the OCJ and TRAP positive osteoclasts as well as nestin and VEGF expression in the subchondral bone compared with MIA rats. Coronal sections of rat joints were stained for Safranin-O fast green showing histological changes in the cartilage and subchondral bone (OCJ), TRAP positive osteoclasts), nestin and VEGF immunoreactivity. Scale bar = 100 µm. Data are presented graphically as mean ± SEM from n = 10 rats/group. *p < 0.05, **p < 0.01, ***p < 0.001, ****p < 0.0001 versus vehicle-treated saline-injected controls. ^+^p < 0.05, ^++^p < 0.01, ^+++^p < 0.001, ^++++^p < 0.0001 versus vehicle-treated MIA-injected OA rats at day 28. ^#^p < 0.05, ^##^p < 0.01, ^###^p < 0.001, ^####^p < 0.0001 versus MIA-injected OA rats at day 14.

**Figure 7:**
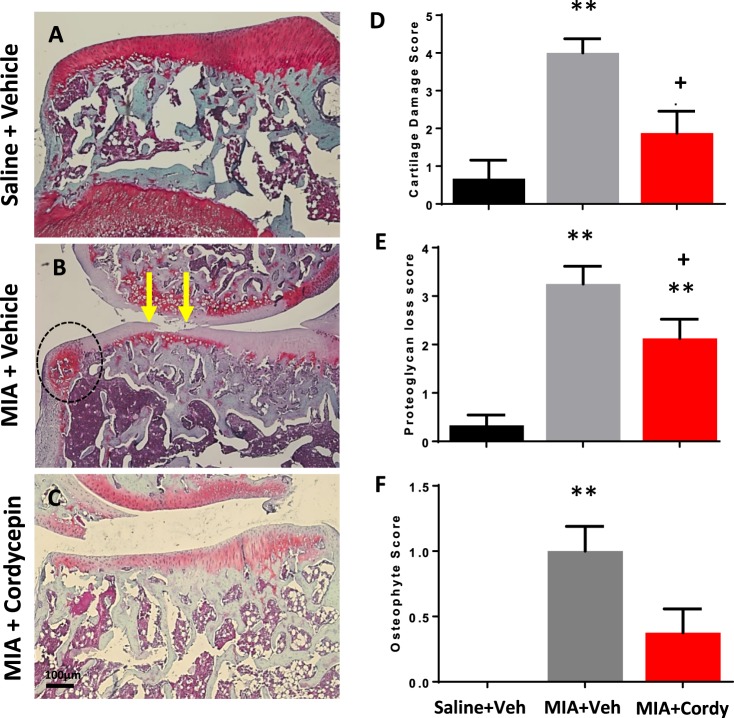
Pre-emptive cordycepin treatment reduces MIA-induced cartilage damage. OA was induced by injecting MIA (1 mg/50 µl) in the left knee joints on day 0. At day 14, cordycepin (Cordy; 8 mg/kg, orally, every other day) or vehicle (Veh) was administered for a period of 2 weeks, from day 0 until day 14. Saline (50 µl) injected rats were used as non-arthritic controls. Vehicle-treated saline-injected control rats showed smooth cartilage and joint margins with normal chondrocyte distribution and proteoglycan staining (**A**). Increased cartilage loss (**B**; arrows) and osteophyte growth (**B**; circle) at joint margins accompanied with chondrocyte hypocellularity and severe loss of proteoglycan staining was observed in the MIA rats (**B**). Cordycepin-treated MIA rats had reduced cartilage damage (**C**,**D**) and improvement in proteoglycan staining (**C**,**E**). Codycepin treatment did not significantly reduce osteophyte score (**C**,**F**). Data are presented graphically as mean ± SEM from n = 10 rats/group. **p < 0.01 versus vehicle-treated saline-injected controls. ^+^p < 0.05 versus vehicle-treated MIA-injected day 14 OA rats.

**Figure 8:**
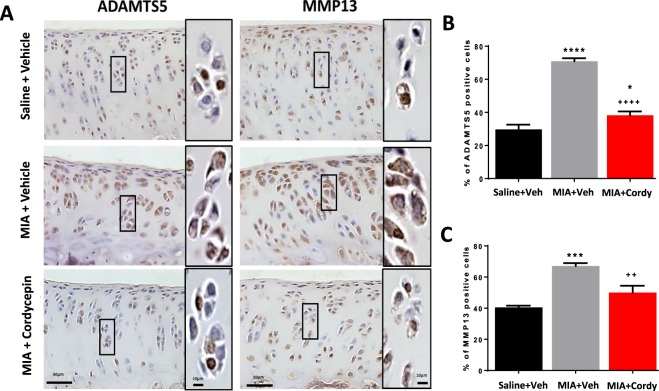
Pre-emptive cordycepin treatment reduces MIA-induced cartilage damage by inhibiting the expression of cartilage proteolytic enzymes. OA was induced by injecting MIA (1 mg/50 µl) in the left knee joints on day 0. At day 14, cordycepin (Cordy; 8 mg/kg, orally, every other day) or vehicle (Veh) was administered for a period of 2 weeks, from day 0 until day 14. Saline (50 µl) injected rats were used as non-arthritic controls. Vehicle-treated saline-injected control rats showed normal chondrocyte distribution and expression of proteolytic enzymes (ADAMTS5 and MMP13). Chondrocyte hypocellularity was observed in the MIA rats with increased expression of proteolytic enzymes by chondrocytes (**A**–**C**). Cordycepin-treated MIA rats showed significant reduction in chondrocyte expression of ADAMTS5 and MMP13. Moreover, following cordycepin treatment, chondrocyte expression of ADAMTS5 and MMP13 appeared to be mostly nuclear and there was a reduction in chondrocyte hypertrophy. Data are presented graphically as mean ± SEM from n = 10 rats/group. *p < 0.05, ***p < 0.001, ****p < 0.0001 versus vehicle-treated saline-injected controls. ^++^p < 0.01, ^++++^p < 0.0001 versus vehicle-treated MIA-injected day 14 OA rats.

**Figure 9:**
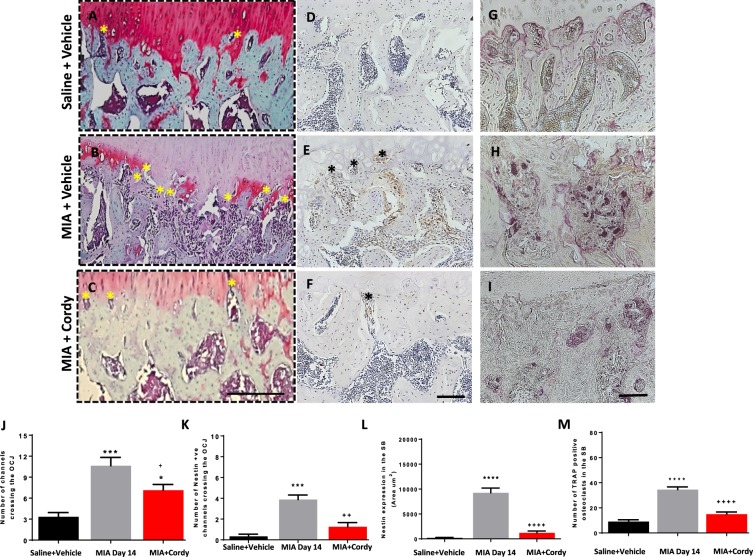
Pre-emptive cordycepin treatment reduces monosodium iodoacetate (MIA)-induced subchondral bone changes. OA was induced by injecting MIA (1 mg/50 µl) in the left knee joints on day 0. Cordycepin (Cordy; 8 mg/kg, orally, every other day) or vehicle (Veh) was administered for a period of 2 weeks from day 0 until day 14. Saline (50 µl) injected rats were used as controls. Cordycepin treatment reduced MIA-induced increase in number of channels crossing the osteochondral junction (OCJ) (**A**–**C** and **J**) and those that were nestin positive (K). MIA-induced increase of nestin expression in the subchondral bone (**D**–**F** and **L**) and tartrate-resistant acid phosphatase (TRAP) positive osteoclasts (**G**–**I** and **M**). Vehicle-treated saline-injected control rats showed fewer number of channels crossing the OCJ and TRAP positive osteoclasts as well as nestin expression in the subchondral bone compared with MIA rats. Safranin-O fast green stained coronal sections of rat joints (**A**–**C**) showing histological changes in the cartilage and subchondral bone. Examples of coronal rat joint sections showing nestin immunoreactivity (**D**–**F**) and TRAP positive osteoclasts (**G**–**I**). Scale bar = 100 µm. Data are presented graphically as mean ± SEM from n = 10 rats/group. *p < 0.05, **p < 0.01, ***p < 0.001, ****p < 0.0001 versus vehicle-treated saline-injected controls. ^+^p < 0.05, ^++^p < 0.01, ^+++^p < 0.001, ^++++^p < 0.0001 versus vehicle-treated MIA-injected OA rats at day 14.

The MIA model of OA was associated with cartilage damage measured as proteoglycan loss, increased chondropathy score and expression of proteolytic enzymes (MMP13 and ADAMTS5). In addition, there was formation of osteophytes and subchondral bone remodelling measured as number of channels crossing the OCJ, expression of TRAP-positive osteoclasts and nestin and VEGF expression in the subchondral bone (Figs 6–9, and Supplementary Fig. 3).

Preventative cordycepin treatment reduced all the measures of MIA-induced cartilage damage (Figs 7 and 8) and subchondral bone remodelling (Fig. 9), but did not significantly alter MIA-induced osteophyte formation (Fig. 7). Therapeutic cordycepin reduced MIA-induced subchondral bone remodelling (Fig. 6), but had no significant effect on MIA-induced cartilage damage and osteophyte formation (Supplementary Fig. 3).

Similar to the MIA model (Figs 2–4, Figs 6–9 and Supplementary Fig. 3), the DMM model was associated with cartilage damage, proteoglycan loss, chondrocyte hypertrophy, increased ADAMTS5 and MMP13 expression in chondrocytes, osteophytosis (size and maturity) and synovial inflammation (Table 1 and Supplementary Fig. 4). Cordycepin treatment reduced all of the above measures of joint pathology in the DMM model (Table 1 and Supplementary Fig. 4). Increased expression of CD68 mRNA was evident in the joints of DMM-operated mice and reduced in the DMM-operated mice treated with cordycepin (Supplementary Fig. 5C). The moderate increase in joint angiogenesis markers (nestin and VEGF) observed in the DMM model (Supplementary Fig. 5A,B) was not significantly affected by cordycepin treatment. The mRNA expression profile of the osteoblast differentiation markers, osterix, RUNX1 and RUNX2 was increased in the joints of DMM mice and reduced with cordycepin treatment (Supplementary Fig. 5). Unlike the MIA model of OA, TRAP positive osteoclasts were not observed in the DMM mice (Supplementary Fig. 4).

### Cordycepin reduces inflammatory transcription in macrophages through inhibiting nuclear localisation of NFĸB

**Figure 10:**
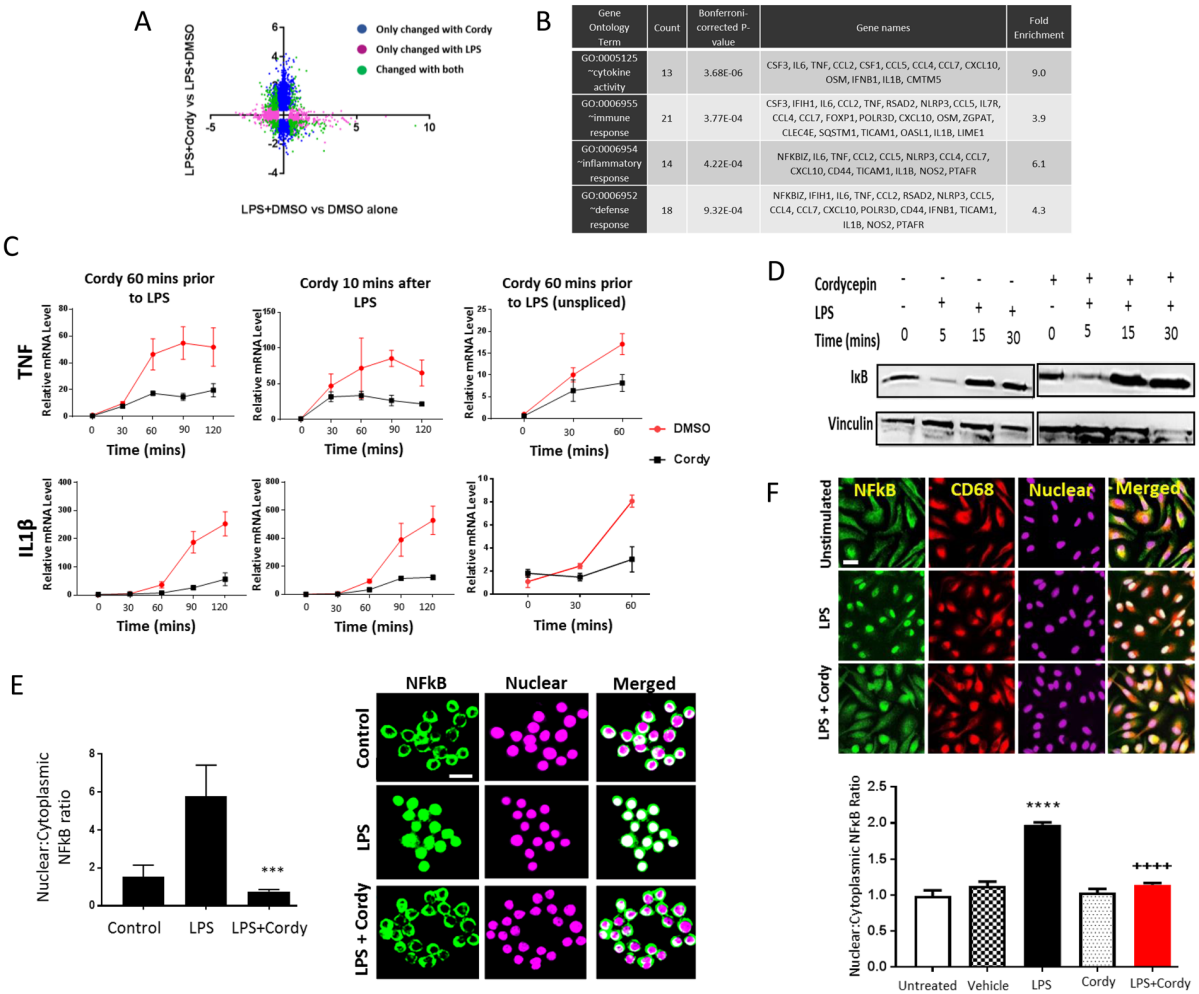
Effects of cordycepin treatment on LPS-stimulated RAW 264.7 macrophages and human monocyte derived macrophages. RAW-264.7 cells were treated with cordycepin (Cordy; 20 μM) either 60 mins prior to or 10 mins after LPS (1 μg/ml) stimulation. DMSO was used as vehicle control. Microarray (A) and gene ontology (**B**) analysis of LPS (60 mins pre-treatment) stimulated cells in the presence and absence of cordycepin. In the microarray data (**A**), the colours indicate statistically significant changes of 2 fold or more. Cordycepin treatment (●) 60 mins before and 10 mins after LPS stimulation (**C**) suppresses LPS-induced activation of inflammatory genes compared to LPS stimulated and DMSO treated cells (●). GAPDH was used as housekeeping gene. Cordycepin (●) prevented the LPS (●) induced increase in unspliced mRNA of TNF and IL1β inflammatory genes (**C**). Cordycepin treatment did not prevent the degradation of IκB as shown in the western blots (**D**). Uncropped images of the blots can be seen in Supplementary Fig. 7. Independent replicates are in Supplementary Fig. 8. At 1 hr, cordycepin reduced nuclear accumulation of NF-ĸB (**E**), reducing nuclear:cytoplasmic NFκB expression ratio (E). Monocytes isolated from peripheral blood of healthy human donors were differentiated into macrophages (**F**). Monocytes grown in the presence of human macrophage colony stimulating factor (MCSF) for 5 days were differentiated into macrophages before stimulation with LPS (100 ng/ml) with and without 20 µM cordycepin for 1 hr. DMSO was used as vehicle control. Cordycepin treatment for 1 hr reduced LPS-induced nuclear accumulation of NF-ĸB in human macrophages, reducing nuclear:cytoplasmic NFκB expression ratio (F). DRAQ5 was used to detect cell nuclei. RAW264.7 data are Mean ± SD of n = 3 biological replicates. Human macrophage data are mean ± SEM of at least n = 3 biological replicates. Scale bar = 10 μm (**E**) and 20 μm (**F)**. ****p < 0.0001 versus vehicle treated group. ^++++^p < 0.0001 versus LPS group.

To elucidate possible direct actions of cordycepin in macrophages we stimulated mouse macrophage cell line RAW264.7 and human blood monocyte derived macrophages with LPS. Microarray analysis of RAW264.7 cells showed that many LPS induced inflammatory mRNAs were repressed by 1 hour pre-treatment with cordycepin (Fig. 10A). There was a significant enrichment of inflammatory genes in the repressed mRNAs (Fig. 10B). Time course analysis for IL1β and TNF mRNA demonstrated effects even when cordycepin was given 10 minutes after LPS (Fig. 10C), indicating a rapid and direct effect on inflammatory gene expression. The unspliced precursors of the inflammatory RNAs were also reduced, suggesting a transcriptional block (Fig. 10C). However, IĸB degradation, which is required for NFĸB to enter the nucleus, was unchanged by cordycepin treatment (Fig. 10D). Despite this normal signal transduction, LPS-induced nuclear localisation of NFĸB was reduced by cordycepin treatment in the RAW264.7 macrophage cell line (Fig. 10E), and in the human monocyte derived macrophages (Fig. 10F). In addition, cordycepin reduced the differentiation of primary human osteoclasts which is also dependent on NFκB (Supplementary Fig. 6). Cordycepin thus appears to affect inflammatory gene transcription through blocking nuclear NFĸB localisation.

### Cordycepin reduces inflammation through polyadenylation inhibition in macrophages

**Figure 11:**
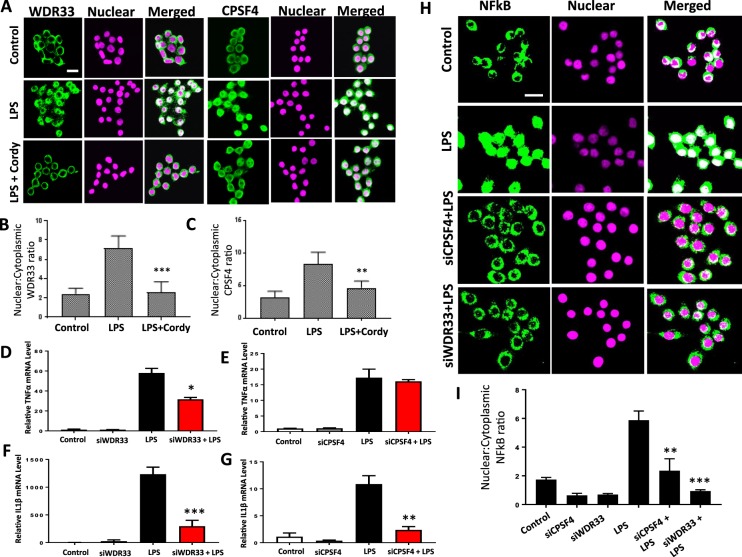
A role for the polyadenylation factors WDR33 and CPSF4 in NFκB mediated transcription. RAW 264.7 macrophages were stimulated with LPS (1 μg/ml) for 10 mins and then treated with 20 μM cordycepin for another 50 mins (**A**–**C**). DMSO was used as vehicle control. Cordycepin treatment reduced the LPS induced nuclear localisation of polyadenylation factors WDR33 (A and B) and CPSF4 (**A**,**C**). siRNA mediated knockdown of polyadenylation factors WDR33 and CPSF4 prevented LPS-induced increase in expression of TNF (**D**,**E**) IL1β (**F**,**G**) and the nuclear accumulation of NFκB (H and I). Mean ± SD of n = 3 biological replicates. *p < 0.05. **p < 0.01, ***p < 0.001, ****p < 0.0001 versus LPS stimulated cells. Scale bar = 10 μm.

Cordycepin is a polyadenylation inhibitor and arrests a complex of factors that include polyadenylation factors such as WDR33 and CPSF4 on the mRNA precursor in polyadenylation reactions in nuclear extracts^16^, however no clear link between polyadenylation factors and inflammation has so far been established. We investigated the effect of cordycepin on the localisation of polyadenylation factors. WDR33 and CPSF4 were predominantly cytoplasmic (Fig. 11) in untreated macrophages. LPS treatment induced localisation of WDR33 and CPSF4 to the nuclei (Fig. 11A), a novel finding that indicates that they are sensitive to inflammatory signalling. Nuclear localisation of polyadenylation factors was reduced by cordycepin (Fig. 11A–C). Cordycepin therefore depletes polyadenylation factors from the nucleus, where they are usually localised during inflammatory gene induction.

To characterise a potential role of polyadenylation factors in NFĸB localisation, we knocked down WDR33 and CPSF4 using siRNA transfection. This knock-down reduced LPS mediated induction of TNF and IL1β mRNAs (Fig. 11D–G) and NFĸB nuclear localisation (Fig. 11H,I), similar to the effect of cordycepin on inflammatory gene expression. Our data therefore indicate that inflammatory gene expression in macrophages is dependent on the presence of high levels of polyadenylation factors in the nucleus.

## Discussion

OA pain is closely associated with synovial inflammation and joint damage^6,46,47^. Inflammation is present at the earliest stages of OA, even before cartilage damage is evident^48,49^. In a significant subset of patients with OA, chronic low-grade inflammation appears to be a major driver of ongoing joint damage^4^. A recent study has demonstrated that OA knees are more sensitive to inflammatory flares, leading to transient increase in pain behaviour and long-term exacerbation of inflammation-induced joint damage^50^. Preventing inflammation may therefore be particularly important in preventing symptoms and long term joint damage in OA. We demonstrate an increased expression of polyadenylation factor CPSF4 associated with synovial inflammation in human OA and that the polyadenylation inhibitor cordycepin sequestered polyadenylation factors in the cytoplasm of human macrophages, reducing nuclear levels of polyadenylation factors. Cordycepin treatment reduced pain behaviour and structural damage in the two rodent models of OA. Our data support a role of polyadenylation in OA progression, inflammatory gene expression and pain.

NF-κB is a central regulator of inflammation, involved in OA pathophysiology and is activated in OA chondrocytes during aging and inflammation^51^. We confirmed that NFκB is overexpressed, and inflammatory markers are increased, in inflamed human OA synovium, as well as in the two rodent models of OA. We used the surgically induced DMM and the chemically induced MIA models of OA, which resemble many features of human OA, pain, synovial inflammation, cartilage damage, osteophytes and subchondral bone changes. Interestingly, these models display varying degrees of inflammation and structural changes, reflecting the heterogeneity of human OA. Oral cordycepin had robust inhibitory effects on pain behaviour whether given therapeutically or preventatively in the rat MIA and the mouse DMM OA models, and reduced inflammatory markers in the synovium, indicating analgesic and anti-inflammatory properties *in vivo*.

Therapeutic cordycepin reduced cartilage damage in the DMM model, but not in the MIA model, despite a significant reduction of cartilage damage by the preventative treatment protocol. The MIA model of OA is a rapidly progressing, severe OA model and at the time when cordycepin was given (day 14 after MIA injection), cartilage damage is advanced and cordycepin treatment did not reverse the existing damage. Our data therefore suggests that cordycepin can prevent, but not repair cartilage damage, and a window of opportunity might exist in early OA during which disease modification is possible. This window was targeted in the more slowly progressing DMM model, in which therapeutic cordycepin (week 14 following DMM model induction) not only reduced OA-associated bone changes, but also reduced cartilage damage. Effects of cordycepin on subchondral bone changes were observed with both therapeutic and preventative treatment regimens. These effects of cordycepin on structural OA (cartilage damage and subchondral bone) may be mediated by suppression of synovial inflammation or direct effects on cartilage or bone. In cultured human chondrocytes and in intervertebral discs, cordycepin had cartilage protective effects^20–23^. In addition tissue culture and animal studies support benefits of cordycepin in preventing bone loss through inhibition of osteoclast differentiation^24–26^, and an osteoprotective effect in osteoporosis^27^. Our data are in keeping with the beneficial effects of intra-articular injection of encapsulated cordycepin on cartilage damage and ADAMTS5 and MMP13 immunoreactivity in the mouse anterior cruciate ligament transection (ACLT) model of OA^28^. However we acknowledge that other potential mechanisms leading to cartilage protection following cordycepin treatment may exist. These may include regulation of autophagy markers and aggrecan neoepitopes generated by aggrecanases and metalloproteases^28^. Our novel finding that orally administered cordycepin reduces synovial inflammation, structural damage and pain in the DMM and MIA models of OA demonstrates that encapsulation is not required to obtain the therapeutic effects of cordycepin.

The mechanisms by which cordycepin attenuates pain behaviour in the models of OA may arise as a result of an inhibition of inflammatory signalling, and/or direct effects on the primary afferent nociceptors. Cordycepin mediated prevention of the activation of mRNA translation in axons^52–55^, which is proposed to occur via cytoplasmic polyadenylation^56,57^ may contribute to the effects of cordycepin on OA pain responses. Local injection of cordycepin into the hindpaw reduced carrageenan-induced and prostaglandin E_2_ -induced hyperalgesia^52,53^, however these studies are complicated by the locally high doses of cordycepin used, which can also inhibit general protein synthesis and may not be specific to polyadenylation of axonal mRNAs^58^.

We report an increase in the synovial expression of the polyadenylation factor CPSF4 associated with inflammation in human OA and that polyadenylation factors undergo nuclear translocation in response to inflammatory signalling in human macrophages, suggesting a role of polyadenylation in inflammatory responses. Our data showing a specific role for polyadenylation factors in the inflammatory process are novel, but are also supported by a previous finding that polyadenylation factor CPSF4 overexpression is required for NFκB mediated transcription in lung cancer cells, suggesting this dependence is not limited to one cell type^59^. Importantly, we show that the polyadenylation inhibitor cordycepin sequesters polyadenylation factors to the cytoplasm of macrophages, lowering nuclear levels of polyadenylation factors.

In combination with our earlier data showing that cordycepin acts intracellularly as cordyTP^19,58^, our data indicate that cordycepin acts on polyadenylation, sequestering polyadenylation factors in an RNA bound complex in the cytoplasm^14–16^, and not as an adenosine receptor agonist^17,18^.

Although the exact role of polyadenylation factors in the inflammatory response and the molecular detail of the mechanisms of action of cordycepin on polyadenylation remain to be elucidated, our data demonstrate cordycepin is orally effective in models of OA pain and indicate that it functions as a polyadenylation inhibitor. We demonstrate that the effects of cordycepin on NFkB mediated transcription is shared between human and rodent macrophages, suggesting that cordycepin will also have the capacity to reduce inflammation in humans. Given that the reported toxicity of cordycepin is low^60–62^, the prospects for clinical application appear excellent. Inflammation is a core component of OA, with subgroups of human knee OA characterized by varying severities of synovial inflammation^4^. For example, generalized nodal OA displays greater synovitis than other OA subtypes^63^. Our data indicate that cordycepin holds promise as the lead compound for a novel class of orally available anti-inflammatory and analgesic drugs, initially for OA patients with high synovial inflammation, and potentially also for other conditions associated with pain and inflammation.

## Supplementary information

Supplementary Info

## Data Availability

All data generated or analysed during this study are included in this published article (and its Supplementary Information files). The microarray dataset has been deposited in the GEO database under accession number GSE126157.

## References

[1] Prieto-Potin I, Largo R, Roman-Blas JA, Herrero-Beaumont G, Walsh DA (2015). Characterization of multinucleated giant cells in synovium and subchondral bone in knee osteoarthritis and rheumatoid arthritis. BMC Musculoskelet Disord.

[2] Ashraf S, Mapp PI, Walsh DA (2010). Angiogenesis and the persistence of inflammation in a rat model of proliferative synovitis. Arthritis Rheum.

[3] Wang X, Hunter DJ, Jin X, Ding C (2018). The importance of synovial inflammation in osteoarthritis: current evidence from imaging assessments and clinical trials. Osteoarthritis Cartilage.

[4] Wyatt LA (2017). Histopathological subgroups in knee osteoarthritis. Osteoarthritis Cartilage.

[5] Cross M (2014). The global burden of hip and knee osteoarthritis: estimates from the global burden of disease 2010 study. Ann Rheum Dis.

[6] Kaukinen P (2016). Associations between MRI-defined structural pathology and generalized and localized knee pain - the Oulu Knee Osteoarthritis study. Osteoarthritis Cartilage.

[7] Yusuf E, Kortekaas MC, Watt I, Huizinga TW, Kloppenburg M (2011). Do knee abnormalities visualised on MRI explain knee pain in knee osteoarthritis? A systematic review. Ann Rheum Dis.

[8] Rigoglou S, Papavassiliou AG (2013). The NF-κB signalling pathway in osteoarthritis. Int J Biochem Cell Biol.

[9] Roman-Blas JA, Jimenez SA (2006). NF-kappaB as a potential therapeutic target in osteoarthritis and rheumatoid arthritis. Osteoarthritis Cartilage.

[10] Boyce BF, Xiu Y, Li J, Xing L, Yao Z (2015). NF-κB-Mediated Regulation of Osteoclastogenesis. Endocrinol Metab (Seoul).

[11] CUNNINGHAM KG, MANSON W, SPRING FS, HUTCHINSON SA (1950). Cordycepin, a metabolic product isolated from cultures of Cordyceps militaris (Linn.) Link. Nature.

[12] Penman S, Rosbash M, Penman M (1970). Messenger and heterogeneous nuclear RNA in HeLa cells: differential inhibition by cordycepin. Proc Natl Acad Sci USA.

[13] Rose KM, Bell LE, Jacob ST (1977). Specific inhibition of chromatin-associated poly(A) synthesis *in vitro* by cordycepin 5′-triphosphate. Nature.

[14] Ryner LC, Manley JL (1987). Requirements for accurate and efficient mRNA 3′ end cleavage and polyadenylation of a simian virus 40 early pre-RNA *in vitro*. Mol Cell Biol.

[15] Zarkower D, Wickens M (1987). Specific pre-cleavage and post-cleavage complexes involved in the formation of SV40 late mRNA 3′ termini *in vitro*. EMBO J.

[16] Shi Y (2009). Molecular architecture of the human pre-mRNA 3′ processing complex. Mol Cell.

[17] Nakamura K (2006). Antitumor effect of cordycepin (3′-deoxyadenosine) on mouse melanoma and lung carcinoma cells involves adenosine A3 receptor stimulation. Anticancer Res.

[18] Won KJ (2009). Cordycepin attenuates neointimal formation by inhibiting reactive oxygen species-mediated responses in vascular smooth muscle cells in rats. J Pharmacol Sci.

[19] Kondrashov A (2012). Inhibition of polyadenylation reduces inflammatory gene induction. RNA.

[20] Li Y (2016). Cordycepin inhibits LPS-induced inflammatory and matrix degradation in the intervertebral disc. PeerJ.

[21] Cao Z (2016). Cordycepin inhibits chondrocyte hypertrophy of mesenchymal stem cells through PI3K/Bapx1 and Notch signaling pathway. BMB Rep.

[22] Ying X (2014). Cordycepin prevented IL-β-induced expression of inflammatory mediators in human osteoarthritis chondrocytes. Int Orthop.

[23] Hu P, Chen W, Bao J, Jiang L, Wu L (2014). Cordycepin modulates inflammatory and catabolic gene expression in interleukin-1beta-induced human chondrocytes from advanced-stage osteoarthritis: an *in vitro* study. Int J Clin Exp Pathol.

[24] Dou C (2016). Cordycepin Prevents Bone Loss through Inhibiting Osteoclastogenesis by Scavenging ROS Generation. Nutrients.

[25] Wang F (2015). Cordycepin prevents oxidative stress-induced inhibition of osteogenesis. Oncotarget.

[26] Kim J, Lee H, Kang KS, Chun KH, Hwang GS (2015). Cordyceps militaris mushroom and cordycepin inhibit RANKL-induced osteoclast differentiation. J Med Food.

[27] Zhang DW, Deng H, Qi W, Zhao GY, Cao XR (2015). Osteoprotective effect of cordycepin on estrogen deficiency-induced osteoporosis *in vitro* and *in vivo*. Biomed Res Int.

[28] Xia C (2017). Photo-crosslinked HAMA hydrogel with cordycepin encapsulated chitosan microspheres for osteoarthritis treatment. Oncotarget.

[29] Imamura K (2015). Suppressing effect of cordycepin on the lipopolysaccharide-induced nitric oxide production in RAW 264.7 cells. Biosci Biotechnol Biochem.

[30] Choi YH, Kim GY, Lee HH (2014). Anti-inflammatory effects of cordycepin in lipopolysaccharide-stimulated RAW 264.7 macrophages through Toll-like receptor 4-mediated suppression of mitogen-activated protein kinases and NF-κB signaling pathways. Drug Des Devel Ther.

[31] Shin S (2009). Role of Cordycepin and Adenosine on the Phenotypic Switch of Macrophages via Induced Anti-inflammatory Cytokines. Immune Netw.

[32] Ashraf S (2014). Augmented pain behavioural responses to intra-articular injection of nerve growth factor in two animal models of osteoarthritis. Ann Rheum Dis.

[33] Glasson SS, Blanchet TJ, Morris EA (2007). The surgical destabilization of the medial meniscus (DMM) model of osteoarthritis in the 129/SvEv mouse. Osteoarthritis Cartilage.

[34] Lee JB (2017). Development of Cordycepin Formulations for Preclinical and Clinical Studies. AAPS PharmSciTech.

[35] Haywood L (2003). Inflammation and angiogenesis in osteoarthritis. Arthritis Rheum.

[36] Altman R (1986). Development of criteria for the classification and reporting of osteoarthritis. Classification of osteoarthritis of the knee. Diagnostic and Therapeutic Criteria Committee of the American Rheumatism Association. Arthritis Rheum.

[37] Sagar DR (2014). Osteoprotegerin reduces the development of pain behaviour and joint pathology in a model of osteoarthritis. Ann Rheum Dis.

[38] Mapp PI (2008). Angiogenesis in two animal models of osteoarthritis. Osteoarthritis Cartilage.

[39] Glasson SS, Chambers MG, Van Den Berg WB, Little CB (2010). The OARSI histopathology initiative - recommendations for histological assessments of osteoarthritis in the mouse. Osteoarthritis Cartilage.

[40] Jackson MT (2014). Depletion of protease-activated receptor 2 but not protease-activated receptor 1 may confer protection against osteoarthritis in mice through extracartilaginous mechanisms. Arthritis Rheumatol.

[41] Ashraf S, Mapp PI, Walsh DA (2011). Contributions of angiogenesis to inflammation, joint damage, and pain in a rat model of osteoarthritis. Arthritis Rheum.

[42] Parfitt AM (1987). Bone histomorphometry: standardization of nomenclature, symbols, and units. Report of the ASBMR Histomorphometry Nomenclature Committee. J Bone Miner Res.

[43] Xu L (2016). The anti-NGF antibody muMab 911 both prevents and reverses pain behaviour and subchondral osteoclast numbers in a rat model of osteoarthritis pain. Osteoarthritis and cartilage / OARS, Osteoarthritis Research Society.

[44] Seegers HC, Hood VC, Kidd BL, Cruwys SC, Walsh DA (2003). Enhancement of angiogenesis by endogenous substance P release and neurokinin-1 receptors during neurogenic inflammation. J Pharmacol Exp Ther.

[45] Caramés B (2012). Autophagy activation by rapamycin reduces severity of experimental osteoarthritis. Ann Rheum Dis.

[46] Neogi T (2016). Association of Joint Inflammation With Pain Sensitization in Knee Osteoarthritis: The Multicenter Osteoarthritis Study. Arthritis Rheumatol.

[47] Sarmanova A (2017). Association between ultrasound-detected synovitis and knee pain: a population-based case-control study with both cross-sectional and follow-up data. Arthritis Res Ther.

[48] Atukorala I (2016). Synovitis in knee osteoarthritis: a precursor of disease?. Ann Rheum Dis.

[49] Felson DT (2016). Synovitis and the risk of knee osteoarthritis: the MOST Study. Osteoarthritis Cartilage.

[50] Ashraf, S., Mapp, P. I., Shahtaheri, S. M. & Walsh, D. A. Effects of carrageenan induced synovitis on joint damage and pain in a rat model of knee osteoarthritis. *Osteoarthritis Cartilage*, 10.1016/j.joca.2018.07.001 (2018). 10.1016/j.joca.2018.07.001 30031926

[51] Saito T, Tanaka S (2017). Molecular mechanisms underlying osteoarthritis development: Notch and NF-κB. Arthritis Res Ther.

[52] Ferrari LF, Bogen O, Chu C, Levine JD (2013). Peripheral administration of translation inhibitors reverses increased hyperalgesia in a model of chronic pain in the rat. J Pain.

[53] Ferrari LF, Araldi D, Levine JD (2015). Distinct terminal and cell body mechanisms in the nociceptor mediate hyperalgesic priming. J Neurosci.

[54] Barragán-Iglesias P (2018). Inhibition of Poly(A)-binding protein with a synthetic RNA mimic reduces pain sensitization in mice. Nat Commun.

[55] Hirai T (2017). Aberrant plasticity of peripheral sensory axons in a painful neuropathy. Sci Rep.

[56] Bogen O, Alessandri-Haber N, Chu C, Gear RW, Levine JD (2012). Generation of a pain memory in the primary afferent nociceptor triggered by PKCε activation of CPEB. J Neurosci.

[57] Charlesworth A, Meijer HA, de Moor CH (2013). Specificity factors in cytoplasmic polyadenylation. Wiley Interdiscip Rev RNA.

[58] Wong YY (2010). Cordycepin inhibits protein synthesis and cell adhesion through effects on signal transduction. J Biol Chem.

[59] Yi C (2016). Cleavage and polyadenylation specific factor 4 targets NF-κB/cyclooxygenase-2 signaling to promote lung cancer growth and progression. Cancer Lett.

[60] Rodman LE (1997). Toxicity of cordycepin in combination with the adenosine deaminase inhibitor 2′-deoxycoformycin in beagle dogs. Toxicol Appl Pharmacol.

[61] Aramwit P, Porasuphatana S, Srichana T, Nakpheng T (2015). Toxicity evaluation of cordycepin and its delivery system for sustained *in vitro* anti-lung cancer activity. Nanoscale Res Lett.

[62] Ma L, Zhang S, Du M (2015). Cordycepin from Cordyceps militaris prevents hyperglycemia in alloxan-induced diabetic mice. Nutr Res.

[63] Kortekaas MC, Kwok WY, Reijnierse M, Huizinga TW, Kloppenburg M (2013). In erosive hand osteoarthritis more inflammatory signs on ultrasound are found than in the rest of hand osteoarthritis. Ann Rheum Dis.

