# Supplementary material for: The polyadenylation inhibitor cordycepin reduces pain, inflammation and joint pathology in rodent models of osteoarthritis
Source: Sci Rep. 2019 Mar 18;9:4696. doi: 10.1038/s41598-019-41140-1 (PMC6423048; doi:10.1038/s41598-019-41140-1)
Supplement: Supplementary file 1 — Supplementary Info [file 41598_2019_41140_MOESM1_ESM.docx]

**The polyadenylation inhibitor cordycepin reduces pain, inflammation and joint pathology in rodent models of osteoarthritis**

**Authors:** Sadaf Ashraf ^1, 4,^, Masar Radhi ^1, 4^, Peter Gowler ^2, 4^, James J Burston ^2, 4^, Raj D Gandhi ^1^ , Graeme J Thorn ^1^, Anna M Piccinini ^1^, David A Walsh ^3, 4, 5^, Victoria Chapman ^2,4,5, *^, Cornelia H de Moor ^1, 4, *^

**Supplementary File**

**
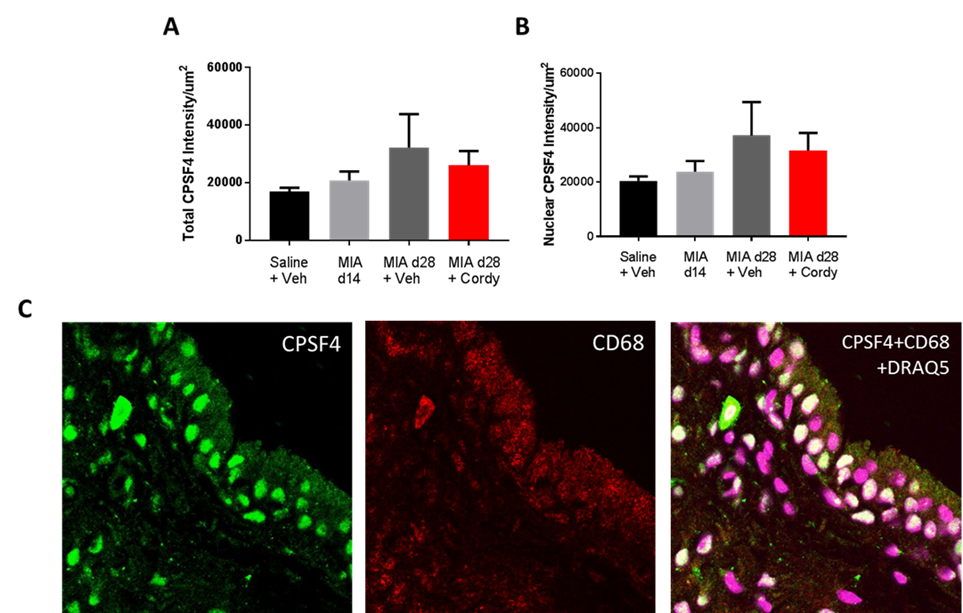
**

**Supplementary Figure 1:** Synovial CPSF4 expression in the MIA model of OA.

OA was induced on day 0 by injecting MIA (1mg/50µl) in the left knee joints of male Sprague Dawley rats. Cordycepin (Cordy; 8mg/kg, orally, every other day) or vehicle (Veh) was administered for a period of 2 weeks, starting at day 14 until day 28. Saline (50µl) injected rats were used as controls. Synovial immunofluorescence staining of the polyadenylation marker CPSF4 (A-C) and the macrophage marker CD68 (C) was conducted on synovial sections from n=10 rats/group. DRAQ5 was used as a nuclear marker. CPSF4 expression was localised to CD68 positive macrophages but the total (A) or the nuclear (B) expression levels did not significantly change in the MIA model of OA and were not affected by cordycepin treatment.

| **Gene** | **Forward Primer Sequence (5’-3’)** | **Reverse Primer Sequence (5’-3’)** |
| --- | --- | --- |
| CD68 | TCCAAGATCCTCCACTGTTG | ATTTGAATTTGGGCTTGGAG |
| IL1β | AGATGAAGGGCTGCTTCCAAA | GGAAGGTCCACGGGAAAGAC |
| IL1β unspliced | TGTCTTTCCCGTGGACCTTC | ACTTGAGGGGCGTTGAGTT |
| TNF | CTATGGCCCAGACCCTCACA | CCACTTGGTGGTTTGCTACGA |
| TNF unspliced | ACACTGACTCAATCCTCCCC | AGCCTTGTCCCTTGAAGAGA |
| Nestin | AGAGAGGGGACCTGGAACAT | CATCCTCCCATTCACTTGCT |
| VEGF | GGAGATCCTTCGAGGAGCACTT | GGCGATTTAGCAGCAGATATAAGAA |
| PCNA | TGCTCTGAGGTACCTGAACT | TGCTTCCTCATCTTCAATCT |
| MYC | TGGAGGAGACATGGTGAACC | AACCGCTCCACATACAGTCC |
| Osterix | ATGGCGTCCTCTCTGCTTG | TGAAAGGTCAGCGTATGGCTT |
| RUNX1 | CAGGCAGGACGAATCACACT | CTCGTGCTGGCATCTCTCAT |
| RUNX2 | GACTGTGGTTACCGTCATGGC | ACTTGGTTTTTCATAACAGCGGA |
| RPL28 | TACAGCACGGAGCCAAATAA | ACGGTCTTGCGGTGAATTAG |
| CPSF4 | GACTGCCCTTGGTATGACCG | CGGGGCTCTCTGCTTTGTTG |
| WDR33 | TTGGACAGTGCACAGAAAGC | TTCGAGCATCCAGAGACTCC |

**Supplementary Table 1:** Mouse primers used for qRT-PCR.

**
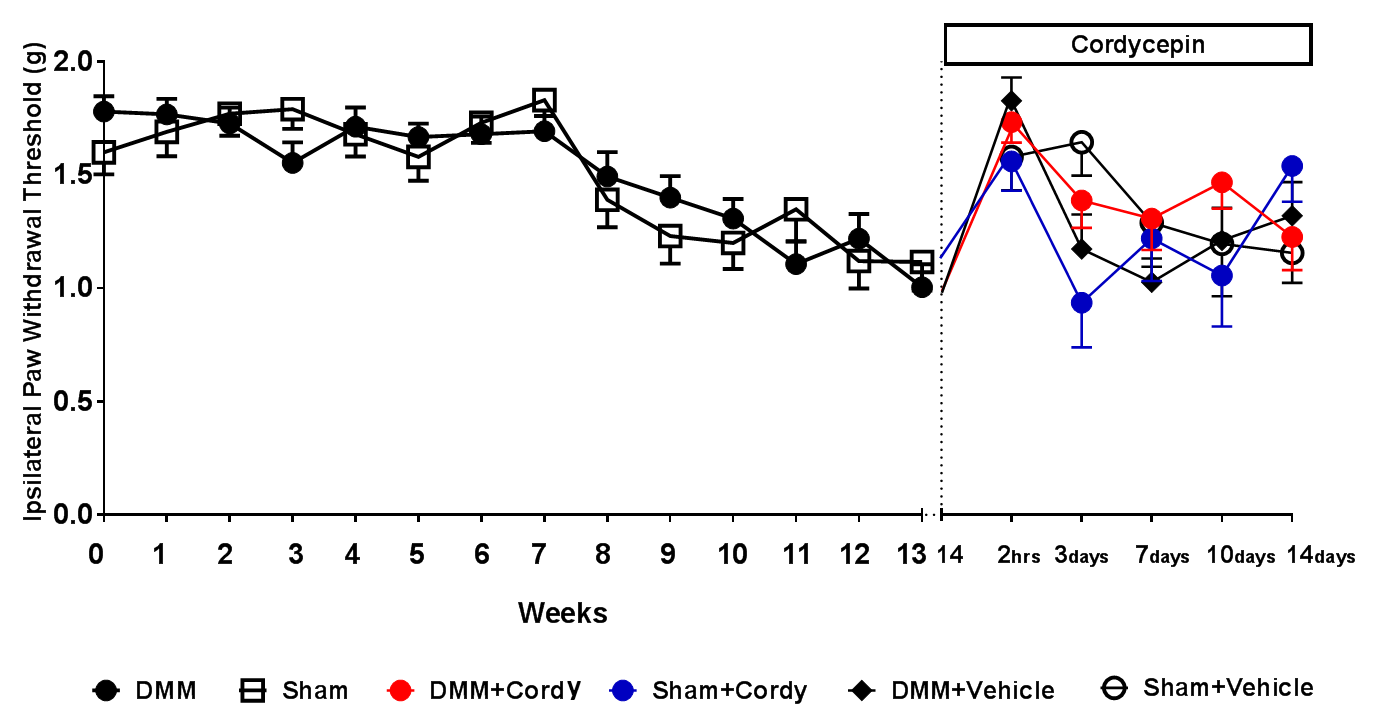
**

**Supplementary Figure 2:** Ipsilateral paw withdrawal threshold is not altered in mice following medial meniscal displacement surgery.

Osteoarthritis (OA) was induced surgically by displacement of the medial meniscus (DMM) in male C57BL/6 mice. From week 14 to 16 mice were orally gavaged every other day with 200µl cordycepin (8mg/kg) or vehicle (23% propylene glycol [PPG] in distilled water) for a period of 2 weeks. Sham-operated mice were used as control in which the ligament was visualised but not transected.


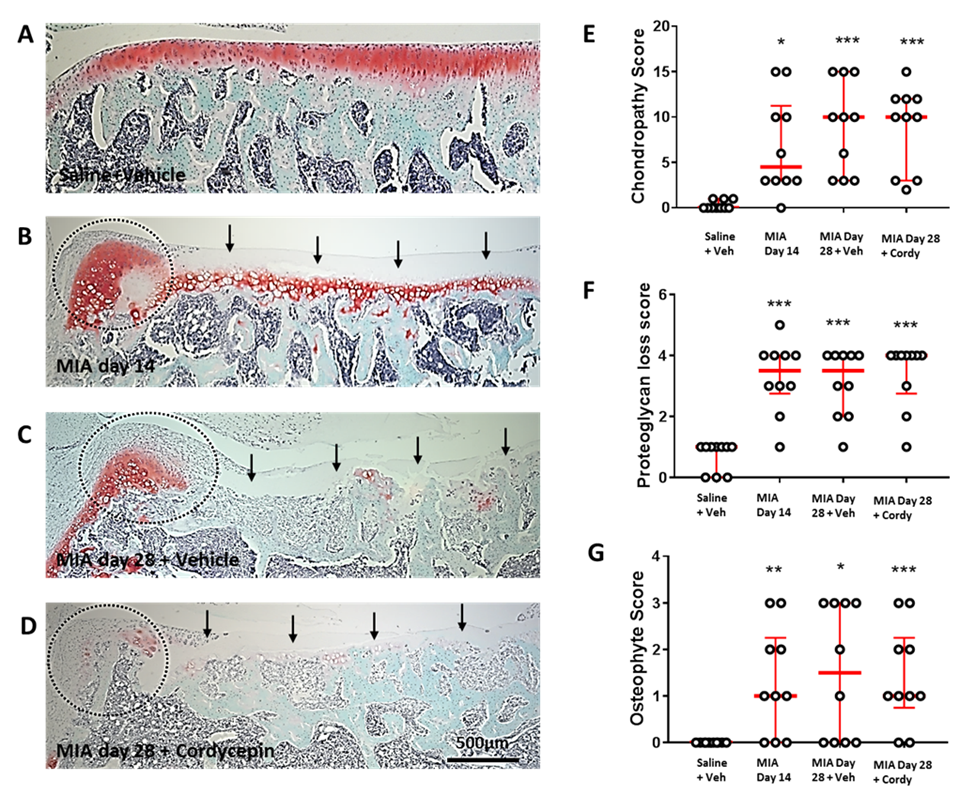


**Supplementary Figure 3:** Cordycepin treatment does not alter monosodium iodoacetate (MIA)-induced cartilage pathology.

OA was induced by injecting MIA (1mg/50µl) in the left knee joints on day 0. At day 14, cordycepin (COR; 8mg/kg, orally, every other day) or vehicle (Veh) was administered for a period of 2 weeks until day 28. Saline (50µl) injected rats were used as controls. Vehicle-treated saline-injected control rats showed smooth cartilage and joint margins with normal chondrocyte distribution and proteoglycan staining (A). Increased cartilage loss (black arrows) and osteophyte growth (circle) at joint margins accompanied with chondrocyte hypocellularity and severe loss of proteoglycan staining was observed as MIA-induced OA progressed from day 14 to day 28 (B, C, and E-G). Rats with established MIA-induced pathological changes at day 14 when treated with cordycepin for a period of 2 weeks however did not show an improvement in cartilage damage, proteoglycan loss or osteophyte size (D and E-G). Data are median±IQR from n=10 rats/group. *p<0.05, **p<0.01, ***p<0.001 versus vehicle-treated saline-injected controls.

**
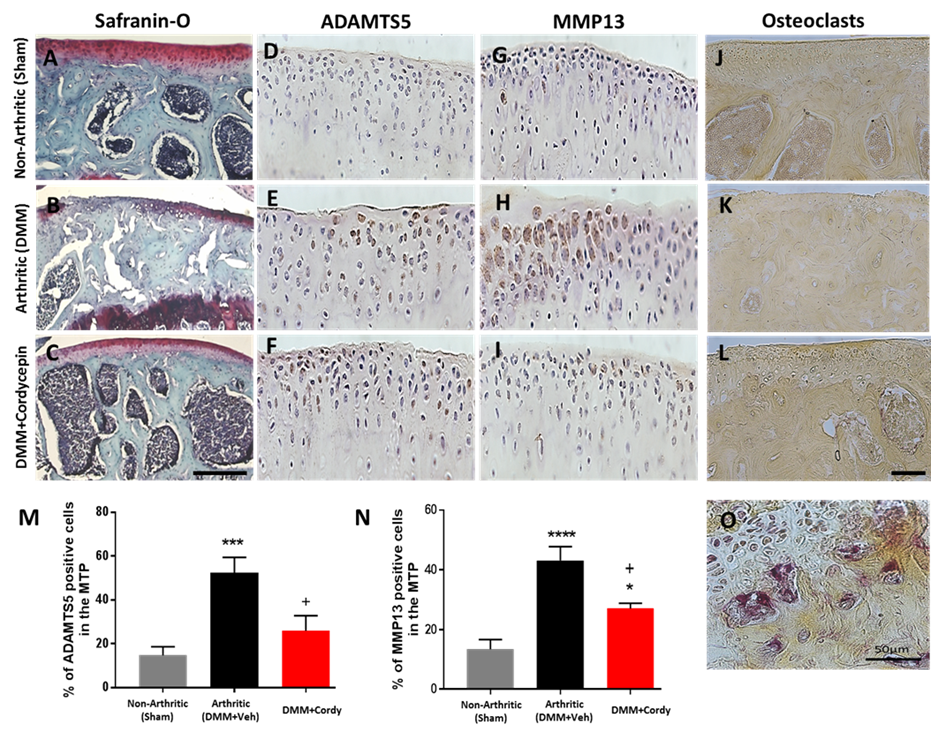
**

**Supplementary Figure 4:** Cordycepin treatment reduces DMM-induced cartilage damage by inhibiting the expression of cartilage proteolytic enzymes.

Non-arthritic (sham) mice showed smooth cartilage and joint margins with normal chondrocyte distribution and proteoglycan staining (A). A mostly nuclear expression of A disintegrin and metalloproteinase with thrombospondin motifs (ADAMTS)-5 and matrix metalloproteinase (MMP)-13 was observed in some of the cartilage chondrocytes of non-arthritic mice (D and G). Cartilage damage, chondrocyte hypertrophy and proteoglycan loss was observed 16 weeks post DMM surgery (B). ADAMTS5 and MMP13 positive chondrocytes also increased in the DMM group compared with the non-arthritic sham operated controls (E, H, M and N). Hypertrophic chondrocytes seemed to have higher expression of ADAMTS5 and MMP13. Cordycepin-treated DMM mice had reduced cartilage damage, improvement in proteoglycan staining (C) and chondrocyte cellularity, accompanied with a significant reduction in chondrocyte expression of ADAMTS5 and MMP13 (F, I, M and N). Moreover, following cordycepin treatment, chondrocyte expression of ADAMTS5 and MMP13 appeared to be mostly nuclear and there was a reduction in chondrocyte hypertrophy. Following DMM-surgery, increased subchondral bone thickening was observed (B) compared to non-arthritic sham operated mice (A) and no TRAP positive osteoclasts were present in the subchondral bone (J-L), however TRAP positive osteoclasts were seen in the bone growth plate (O). Sagittal Safranin-O fast green stained mice joint sections (A-C) showing histological changes in the cartilage. Immunostained sections showing ADAMTS5 positive (D-F) and MMP13 positive (G-I) chondrocytes in the cartilage. Data are presented graphically as mean±SEM. *p<0.05, **p<0.01, ***p<0.001, ****p<0.0001 versus non-arthritic sham operated controls. +p<0.05 versus arthritic (DMM) group.


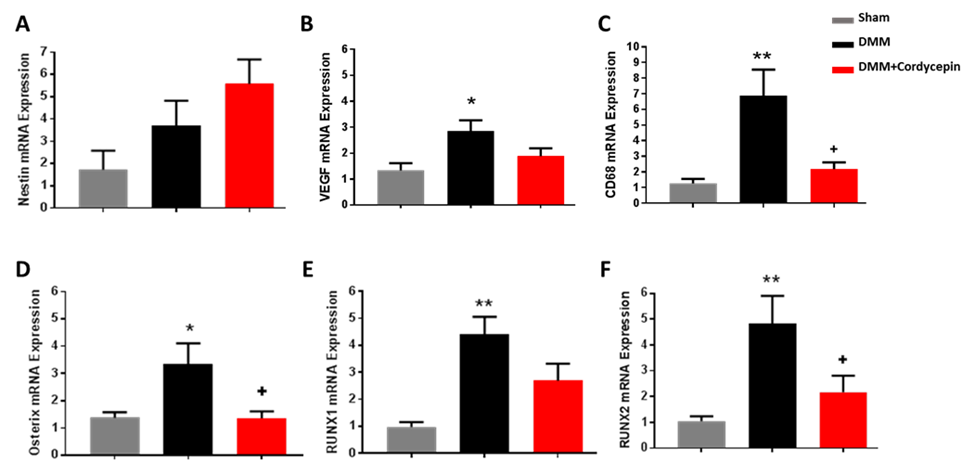


**Supplementary Figure 5:** Cordycepin treatment reduces DMM-induced increase in knee joint osteoblast markers.

In the DMM model of OA, cordycepin treatment had no effect on joint angiogenesis (A; nestin and B; VEGF mRNA expression levels) but it did reduce joint inflammation (C; CD68 mRNA expression levels). DMM model of OA was accompanied with an increase in joint mRNA expression of several osteoblast differentiation markers (D; osterix, E; RUNX1 and F; RUNX2) compared with non-arthritic sham operated mice. Following cordycepin treatment in the DMM mice, a reduction in the osteoblast differentiation markers was seen (D-F). Data are mean±SEM. *p<0.05, **p<0.01, ***p<0.001, ****p<0.0001 versus non-arthritic sham operated controls. +p<0.05 versus arthritic (DMM) group.

**
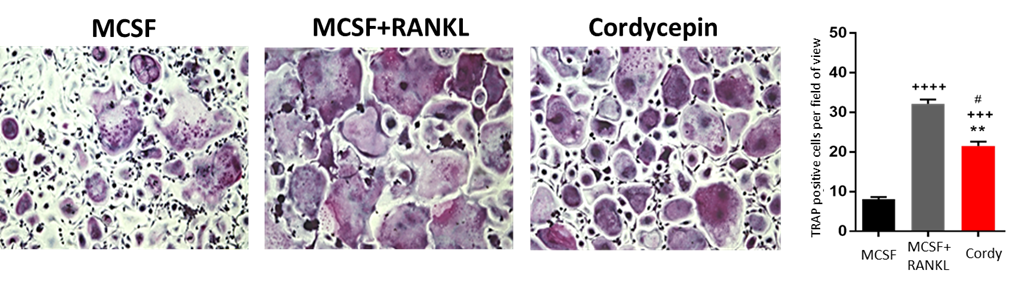
**

**Supplementary Figure 6.** Effect of cordcepin on human osteoclast differentiation.

Monocytes isolated from peripheral blood of healthy human donors were differentiated into osteoclast. Monocytes grown in the presence of macrophage colony stimulation factor (MCSF) and human receptor activator of NF-ĸB ligand (RANKL) for 14 days differentiated into osteoclasts as identified by TRAP staining and had greater number of osteoclasts compared with MCSF only treated monocytes. Cordycepin (20µM) treatment prevented osteoclast differentiation. Cells were seeded and grown on glass coverslips within a 24-well culture plates and for quantification of TRAP positive cells five random fields of view were counted per coverslip using four coverslips per condition. Cells that stained positive for TRAP and had three or more nuclei were counted. Human osteoclast data are mean ±SEM of at least n=3 biological replicates. *p<0.05, ** p<0.01, *** p<0.001, ****p<0.0001 versus MCSF+RANKL group. +++p<0.001, ++++p<0.0001 versus MCSF group.


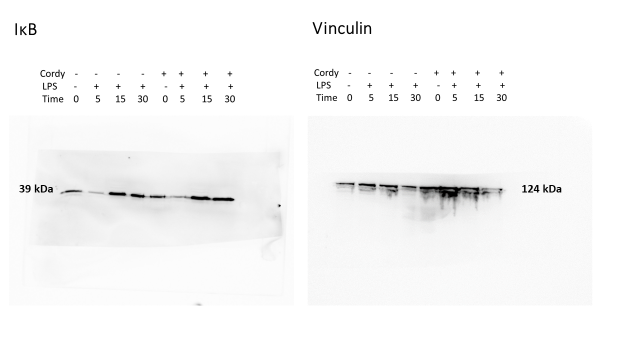


**Supplementary Figure 7:** Uncropped image of the western blot used in the manuscript.

**
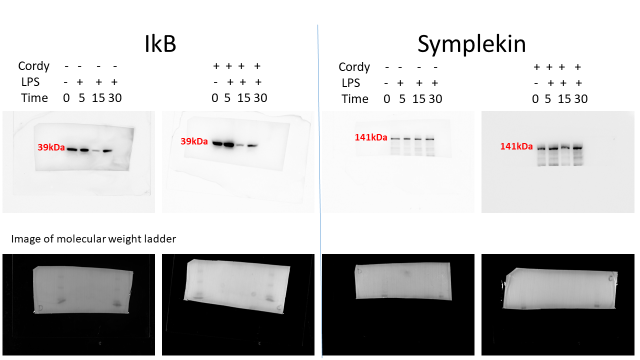
**

**Supplementary Figure 8.** Un-cropped western blots highlighting the expression of IkB and loading control symplekin.
